# Transcriptional Consequence and Impaired Gametogenesis with High-Grade Aneuploidy in *Arabidopsis thaliana*

**DOI:** 10.1371/journal.pone.0114617

**Published:** 2014-12-16

**Authors:** Kuan-Lin Lo, Long-Chi Wang, I-Ju Chen, Yu-Chen Liu, Mei-Chu Chung, Wan-Sheng Lo

**Affiliations:** 1 Institute of Plant and Microbial Biology, Academia Sinica, Taipei, Taiwan; 2 Biotechnology Center, National Chung-Hsing University, Taichung, Taiwan; University of Science and Technology of China, China

## Abstract

Aneuploidy features a numerical chromosome variant that the number of chromosomes in the nucleus of a cell is not an exact multiple of the haploid number, which may have an impact on morphology and gene expression. Here we report a tertiary trisomy uncovered by characterizing a T-DNA insertion mutant (*aur2-1*/+) in the Arabidopsis (*Arabidopsis thaliana*) *AURORA2* locus. Whole-genome analysis with DNA tiling arrays revealed a chromosomal translocation linked to the *aur2-1* allele, which collectively accounted for a tertiary trisomy 2. Morphologic, cytogenetic and genetic analyses of *aur2-1* progeny showed impaired male and female gametogenesis to various degrees and a tight association of the *aur2-1* allele with the tertiary trisomy that was preferentially inherited. Transcriptome analysis showed overlapping and distinct gene expression profiles between primary and tertiary trisomy 2 plants, particularly genes involved in response to stress and various types of external and internal stimuli. Additionally, transcriptome and gene ontology analyses revealed an overrepresentation of nuclear-encoded organelle-related genes functionally involved in plastids, mitochondria and peroxisomes that were differentially expressed in at least three if not all Arabidopsis trisomics. These observations support a previous hypothesis that aneuploid cells have higher energy requirement to overcome the detrimental effects of an unbalanced genome. Moreover, our findings extend the knowledge of the complex nature of the T-DNA insertion event influencing plant genomic integrity by creating high-grade trisomy. Finally, gene expression profiling results provide useful information for future research to compare primary and tertiary trisomics for the effects of aneuploidy on plant cell physiology.

## Introduction

Aneuploid cells contain a chromosome variant with chromosome number other than a multiple of the basic (monoploid) chromosome number (x), which may alter large-scale gene expression leading to cellular malfunction and diseases due to genomic imbalance [1], [2]. Aneuploidy results from failure of the chromosomes or chromatids to separate properly to opposite poles during meiosis or mitosis. Trisomy (2n = 2x+1) is a type of aneuploidy that involves an extra copy of a particular chromosome rather than the normal number of 2 [2]. In humans, trisomy causes severe developmental defects in fetuses, and only newborns with a few types of trisomy can survive [3]. The most commonly known disease caused by trisomy in humans is Down syndrome, which affects individuals with an extra chromosome 21 in whole or in part [4].

Recent work has suggested that the unbalanced genome in aneuploidy may result in loss of genomic integrity and epigenetic changes in many organisms [5]–[7]. Genes on the aneuploid chromosomes frequently show a dosage compensation effect that probably helps mitigate the harmful consequences of genomic imbalance. Despite the detrimental effects in cells, aneuploidy sometimes provides a means for adapting to selective pressure [8]. Aneuploidy is highly associated with poor prognosis, high malignancy and increased drug resistance in tumorgenesis [9]–[11]. Studies of humans, mice and yeast suggest that an abnormality in chromosome number or structure alters cellular physiology by resulting in changes in genome stability, imbalanced protein homeostasis and numerous dysfunctional growth characteristics (see reviews in [10], [12], [13]).

Whole-chromosome aneuploidy, which can be used to examine the physiological effects in aneuploid cells, is relatively easy to generate and maintain than high-grade aneuploidy [5], [14]. However, the molecular mechanisms and transcriptional signatures underlying the organismic physiological alterations of aneuploidy are not fully understood [14]. Considering the diverse phenotypes induced by different degrees of aneuploidy, whether the knowledge from the studies of simple aneuploidy can be informative for understanding the role of complicated aneuploidy in cancer and genetic diseases is unclear [15]. Thus, further studies of the cellular physiology of aneuploidy will increase our understanding of the causal effects and consequences of genomic imbalance in eukaryotic cells.

Plants are more tolerant than animals to genomic imbalance caused by aneuploidy [1] and can be manipulated to generate different karyotypes for viable individuals [1], [12]. Thus, research of plants has provided an excellent opportunity to study the transcriptional consequence of different types of aneuploidy. Trisomy in the 5 chromosomes in Arabidopsis (*Arabidopsis thaliana*) has been described, and different types of trisomy can be distinguished morphologically as compared with diploid (2n = 2x) plants [1], [16]. Different types of trisomy have been documented in plants primarily by genetic, cytogenetic and morphological analyses. Trisomic plants produce offspring with various karyotypes; most of the progeny are diploid and some are trisomic. Tetrasomics (2n = 2x+2) are extremely rare, and some types of tetrasomy have never been observed [16]. Tertiary trisomy (2n = 2x+T) is present in individuals carrying an extra copy of a chromosome consisting of portions of 2 non-homologous chromosomes in the genome [2].

Transgenic plants carrying T-DNA insertions have been widely used in plant research for forward and reverse genetics to generate mutants for functional studies [17]. A T-DNA inserted in genomic sequence should disrupt or reduce the function of a gene if insertion is near or within the coding sequence. However, several reports have documented T-DNA insertions resulting in or associated with chromosomal abnormalities such as sequence deletion, duplication, the addition of unknown filler sequences, or chromosome rearrangement in plants [18], [19]. We have limited information about the physiological consequences of numerical abnormalities in chromosomes resulting from T-DNA insertions. A previous study demonstrated that a transgenic tobacco line with a T-DNA insertion generated trisomic and tetrasomic offspring [6], [7]. However, the cause of the aneuploid progeny was unclear.

In this study, we uncovered an example of high-grade aneuploidy (tertiary trisomy 2) when characterizing a T-DNA insertion allele in the Arabidopsis *AURORA2* locus at chromosome 2. The *aur2-1* mutation was associated with defective development in male and female gametophytes and showed unusual genetic transmission. Additionally, we found more genes on the non-triplicated chromosomes that were mis-expressed in the high-grade trisomy than the primary trisomy 2; the former carried a chromosomal translocation that would have a significant impact on cell physiology. Finally, transcriptome and gene ontology (GO) analysis revealed overrepresentation of genes involved in the stress response, plastids and mitochondria in two primary and one tertiary trisomics, which supported increased energy demand and cellular metabolism as a common response to different types of stress resulting from genomic imbalance in aneuploidy [20].

## Materials and Methods

### Plant materials and growth conditions

The wild-type *A. thaliana* ecotype Columbia-0 (Col-0); two T-DNA insertion mutants of *AUR2*, Wisconsin DsLox T-DNA line (WisDsLox_368B03; CS853125) and GABI-Kat line (GK403B02; CS438606); and the *quartet* mutant, *qrt2-1* (Landsberg *erecta*, L*er*, CS8051) were obtained from the Arabidopsis Biological Resource Center (ABRC) or Nottingham Arabidopsis Stock Centre (NASC). We designated the T-DNA insertion alleles in *AUR2* (At2g25880) as *aur2-1* (WisDsLox_368B03) and *aur2-2* (GK403B02) according to a previous study [21]. The *aur2-1* line was designated *AAa*, where “*a*” indicates the T-DNA insertion at *AUR2*, and the diploid and trisomic plants derived from the *aur2-1* line but lacking a T-DNA insertion were designated *AA* and *AAA,* respectively. The plants were cultivated in growth chambers under long-day conditions (16 h-light/8 h-dark) at 22°C with light intensity 100–150 µE m^−2^ s^−1^. Primers used to genotype the *aur2-1* allele were RP, 5’-cgaaatcgcttgtagtccatc-3’; LP, 5’-ggcaatggagaagagtgtcac-3’; and BP, 5’-aacgtccgcaatgtgttattaagttgtc-3’.

### Pollen viability and *in* *vitro* germination assays

Staining for pollen viability was as described previously [22] with minor modifications. Briefly, fluorescein diacetate (FDA) (Sigma) was dissolved in 0.5% (w/v) acetone and diluted (1∶49) in 10% sucrose as a working solution. Mature pollen grains were incubated with FDA staining solution for 15 min in the dark before observation under a fluorescence microscope (Axio Scope A1, Zeiss). The number of viable and non-viable pollen grains was counted by use of ImageQuant TL (GE Healthcare). The *in* *vitro* germination assay was performed as described [23]. The morphologic features of germinating pollen and pollen tube elongation were examined and scored under a microscope. The length of the elongated pollen tube was measured by use of ImageJ (http://rsbweb.nih.gov/ij/).

### Morphologic analysis of pollen grains, ovules, embryogenesis and seed formation

For analysis of tetrads in the *qrt2-1*/*qrt2-1* and *aur2-1*/+ *qrt2-1*/*qrt2-1* plants, mature pollen grains from open flowers were transferred to filtered water and observed on slides under a microscope. Mature pollens treated with 4′,6-diamidino-2-phenylindole (DAPI) (Sigma) were placed on a slide and examined under a florescence microscope as described [24]. To observe the phenotype of mature ovules, we emasculated flowers at stage 12 [25], then fixed carpels for 2 d to ensure that the maximum number of ovules reached maturity. To observe the developing embryos, flowers were manually pollinated and siliques were fixed 6 or 10 d after pollination (DAP). Carpels or siliques were fixed and cleared as described [26]. Ovules or developing seeds were mounted in the clearing solution and examined under a microscope with differential interference contrast (DIC) optics. To evaluate fertilization and seed formation, self- or manually pollinated flowers were further grown for 10 d. Siliques were collected and dissected to score seed sets and unfertilized ovules.

### Chromosome preparation for cytological analyses

The chromosome spreads for DAPI staining in pollen mother cells (PMCs) and shoot apical meristem (SAM) cells were performed as described [27], [28]. PMC and SAM cells were fixed with fresh Carnoy fixative [ethanol-acetic acid (3∶1, v/v)] at 4°C overnight and stored at −20°C. The tissues were washed once with filtered water and twice with citrate buffer (10 mM sodium citrate, pH 4.8) and incubated in digestion solution [0.02% (w/v) cellulase ONOZUKA R-10 and 0.2% (w/v) Macerozyme R-10 (both from Yakult, Japan) in citrate buffer] at 37°C for 1 h. Then tissues were washed with filtered water, decomposed in a drop of 60% acetic acid, fixed on slides, and stained with DAPI solution for fluorescence microscopy. Fluorescence *in situ* hybridization (FISH) of mitotic cells in SAM was performed as described [29]. All images were acquired by use of AxioVision software (Zeiss) and analyzed by use of Adobe Photoshop CS2.

### Cryo-scanning electron microscopy (Cryo-SEM)

Fully open flowers were dissected and loaded on stubs, then samples were frozen with liquid nitrogen slush and transferred to a preparation chamber at −160°C. After 5 min, the temperature was raised to −85°C and sublimed for 15 min. After being coated with platinum (Pt) at −130°C, samples were transferred to the cold stage in the SEM chamber and observed at −160°C by use of Cryo-SEM (FEI Quanta 200 SEM/Quorum Cryo System PP2000TR FEI) at 20 KV.

### Comparative genome hybridization with Arabidopsis tilling array (array CGH)

Genomic DNA (gDNA) was extracted from rosette leaves by use of the DNeasy Plant Mini Kit (Qiagen). Affymetrix Arabidopsis Tilling 1.0R arrays were used for hybridization with labeled gDNA prepared from Col-0 and trisomic plants (*AAA* and *AAa*, see above). DNA labeling, hybridization, and detection followed the manufacturer’s instructions (Affymetrix). Probe sequences from the BPMAP specification of the array (At35b-MR_v04-2_TIGRv5) were mapped to the TAIR10 genome by use of BLAT [30]. Intensity normalization involved the AffyTiling package of BioConductor. Probe locations and normalized signals were joined and sorted by use of an in-house perl script. Raw data for the tilling array are available in the Gene Expression Omnibus (GEO) database (http://www.ncbi.nlm.nih.gov/geo/) (accession nos. GSM980297, GSM980298 and GSM980299).

### Whole-genome transcriptome analysis

For expression microarray analysis, samples of total RNA were prepared from 26-d-old plants of the wild type (Col-0), trisomy 2 (*AUR2*, round leaves, *AAA* genome), and tertiary trisomy 2 (*aur2-1*; round leaves, *AAa* genome), respectively. The karyotype and genotype were first scored by leaf morphology (round leaves), then underwent genotyping before sample collection. RNA samples from the rosette leaves of multiple plants (21–35 plants) were extracted by use of the RNeasy Plant Mini Kit (QIAGEN). Florescence labeling, hybridization to probes and detection followed the manufacturer’s instructions (Affymetrix). The sequence annotation for the Affymetrix ATH1 oligos was downloaded from TAIR and processed by use of Excel (Microsoft); probe sets not mapping to two or more gene loci were selected [5]. Sequence-specific probe effects were removed by use of the GC-RMA package from R software [31]. The 14,976 probe sets for genes on chromosomes 1, 4, and 5 and those on chromosome 3 from the 3′ end of At3g20040 (*AUR2*) to the end were selected for model fitting by use of vsn package [32] and the hybridization intensities were then normalized by using the fitted model [5]. Genes on the non-triplicated chromosome with sufficiently strong expression were isolated as described (Figure S4 in S1 File) [5], [14]. Changes in gene expression and gene ontology (GO) analysis involved use of GeneSpring (Agilent) and ArgoGO [33]. The raw data for Affymetrix ATH1 arrays are available in the Gene Expression Omnibus (GEO) database (http://www.ncbi.nlm.nih.gov/geo/) (accession no. GSE54827).

## Results

### Identification of a trisomic Arabidopsis plant with a T-DNA insertion

**Figure 1:**
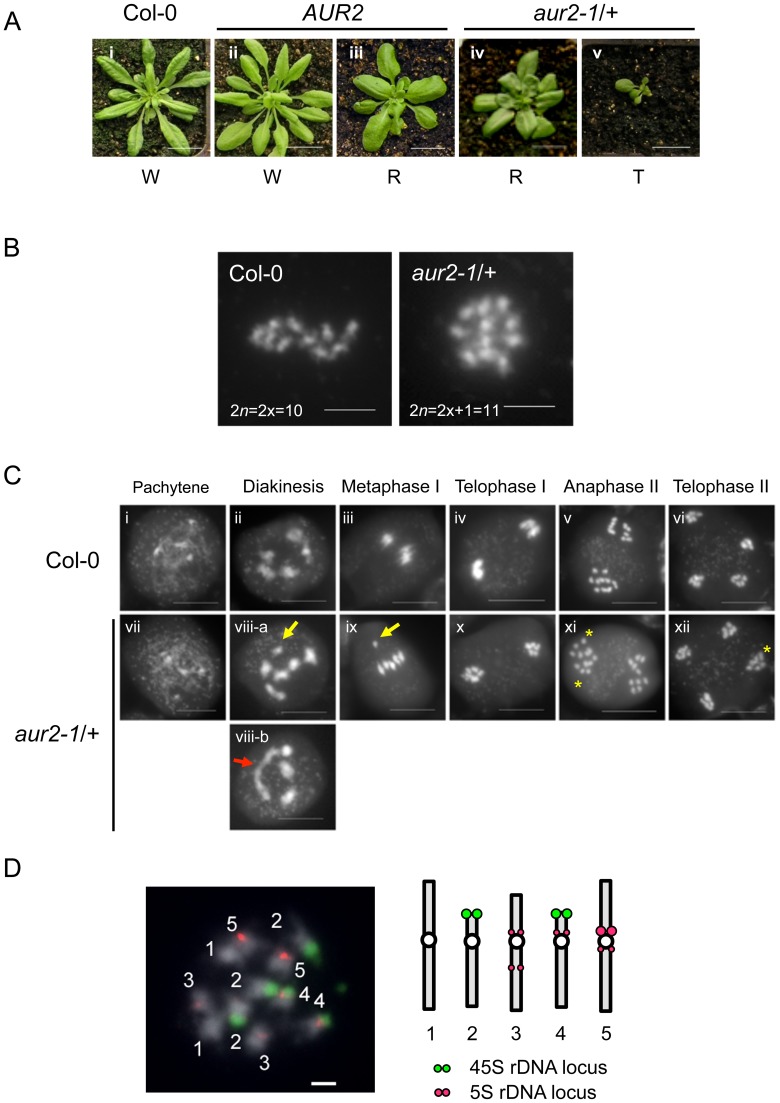
The *aur2-1*/+ mutant is trisomy 2. (**A**) Rosette phenotype of wild type (Col-0) and progeny from self-fertilized *aur2-1*/+. W, wild-type rosette leaves; R, round-shaped rosette leaves; T, tiny rosette leaves. (**B**) Metaphase spreads of the mitotic cells from Col-0 and *aur2-1*/+. (**C**) Meiosis in pollen mother cells of Col-0 and *aur2-1*/+. DAPI-stained chromosomes in different meiotic stages. Yellow arrows, extra univalents; red arrow, pentavalent; yellow stars, extra sister chromatids. (**D**) Fluorescence *in situ* hybridization analysis of *aur2-1*/+. Metaphase spreads of the *aur2-1*/+ mitotic cells probed with FITC-labeled 45S rDNA (green) and rhodamine-labeled 5S rDNA (red). Dashed lines, outline of DAPI-stained chromosome. Scale bar in (**A**) 2 cm; (**B**) 5 µm; (**C**) 10 µm; (**D**) 2 µm.

In this study, we discovered two types of aneuploidy in a heterozygous T-DNA insertional mutant (*aur2-1*/+) (Figures S1A and S1B in S1 File) and its descendants. The proportion of undeveloped seeds in self-fertilized plants was 18.6% and 1.2% for *aur2-1*/+ and the wild type (WT = Col-0), respectively, under the same growth conditions (Table S1 and Figure S1C in S1 File), which suggests defective fertilization or embryo development or both in *aur2-1*/+. The round shape of rosette leaves in the progeny of *aur2-1*/+ is consistent with the description of an aneuploid mutant with trisomy 2 in Arabidopsis [1], [16], [34] (Fig. 1A–iv). Thus, we examined the metaphase chromosome spread of mitotic cells to confirm this possibility. WT cells had 10 chromosomes (2n = 2x), and *aur2-1*/+ cells had 11 (2n = 2x+1) (Fig. 1B). We next analyzed the meiotic chromosome spreads in pollen mother cells (Fig. 1C). The number of DAPI-stained chromosomes differed between WT and *aur2-1*/+ as early as diakinesis stage. Chromosome spreads showed 1 univalent plus 5 bivalents or 1 pentavalent plus 3 bivalents for *aur2-1*/+ but 5 typical bivalents in the WT (Fig. 1C, viii-a and viii-b vs. ii). The WT showed the 5 bivalents aligned along an equatorial plate, whereas an extra univalent out of the metaphase plate appeared in *aur2-1*/+ at metaphase I (Fig. 1C, iii vs. ix). Furthermore, in the WT, 5 pairs of sister chromatids were segregated to each daughter cell, whereas *aur2-1*/+ showed an extra DAPI dot in each nucleus during sporogenesis from anaphase-II and telophase-II to pollen tetrads (Fig. 1C, v–vi vs. xi–xii). Additionally, we analyzed the karyotype of *aur2-1*/+ by using 5S and 45S rDNA probes labeled with different dyes in dicolor FISH (Fig. 1D, right panel) [35]: *aur2-1*/+ showed 11 DAPI-stained chromosomes from shoot apical meristem cells and 3 sets of green florescence dye (45S rDNA) at chromosome 2 (Fig. 1D, left panel). In sum, both the rosette leaf phenotype and cytological studies demonstrated that *aur2-1*/+ is trisomy 2 and the additional chromosome appears to have a functional centromere for chromosome segregation during meiosis and mitosis.

### Genotype and karyotype of *aur2-1*/+ progeny

On genotyping nearly 1,000 plants of the self-fertilized heterozygous *aur2-1*/+ mutant, we did not find any homozygous progeny, which suggests that the *aur2-1* allele may have an unusual inheritance in the trisomic genome. To understand the genetic behavior and genomic constitution of *aur2-1/+*, we performed genotype and karyotype analyses of *aur2-1*/+ progeny. By reciprocal crosses between the *aur2-1*/+ and WT, we found a 25.2% transmission rate of *aur2-1* from *aur2-1*/+ ovules, which was comparable to that of *aur2-1* in F1 progeny of self-fertilized *aur2-1*/+ plants (27.1%); however, we found no *aur2-1* allele in F1 progeny when WT ovules were pollinated with *aur2-1*/+ pollen (Table 1). Therefore, pollen failed to transmit the *aur2-1* allele to offspring and the inheritance of *aur2-1* allele is mainly through maternal transmission.

**Table 1:** Genetic analysis of *aur2-1*/+ inheritance.

Genotype of parents (female×male)	Genotype of F1 progeny			Plants	Plants with an *aur2-1* allele (%)
	*AUR2*	*aur2-1* HZ^a^	*aur2-1* HM^b^		
*aur2-1*/+ self-fertilized	140	52	0	192	27.08
*aur2-1*/+ × Col-0	122	41	0	163	25.15
Col-0 × *aur2-1*/+	115	0	0	115	0

a HZ, heterozygous.

b HM, homozygous.

Because the round leaf phenotype is a morphological characteristic of Arabidopsis trisomy 2 plants, we used it as a reference to visually assess the karyotype of plants [16], [34]. We found 3 major types of *aur2-1*/+ progeny from both self-fertilization and reciprocal crosses: plants carrying a WT *AUR2* with WT leaves (*AUR2*, W) or round leaves (*AUR2*, R) and *aur2-1*/+ plants with round leaves (*aur2-1/+*, R) (Table 2; Fig. 1A); however, we found no *aur2-1*/+ plants with WT leaves. In addition, we found a few plants (7/242) from self-fertilized *aur2-1*/+ with extremely small and tiny (T) round leaves that died before bolting (Table 2, Fig. 1A-v). Tetrasomics grow slowly and show an enhanced phenotype as compared with corresponding trisomics in *Arabidopsis* and other species [16], [36]; thus, the plants with tiny rosette leaves may be tetrasomics.

**Table 2:** Genotype and rosette leaf phenotype in the progeny of self-fertilized and backcrossed *aur2-1*/+.

	*AUR2*			*aur2-1*/+			
Leaf phenotype (genotype)^d^	W^a^ ^(*AA*)^	R^b^ ^(*AAA*)^	T^c^	W^a^ ^(*Aa*)^	R^b^ ^(*AAa*)^	T^c^	Plants
Cross							
*aur2-1*/+ self-fertilized	152	11	0	0	72	7	242
*aur2-1*/+ × Col-0	71	4	0	0	26	0	101

a W, plants with wild-type rosette leaves.

b R, plants with round rosette leaves.

c T, plants with tiny stature and round rosette leaves.

d Predicted genotype.

### Chromosomal translocation in the *aur2-1*/+ trisomy

**Figure 2:**
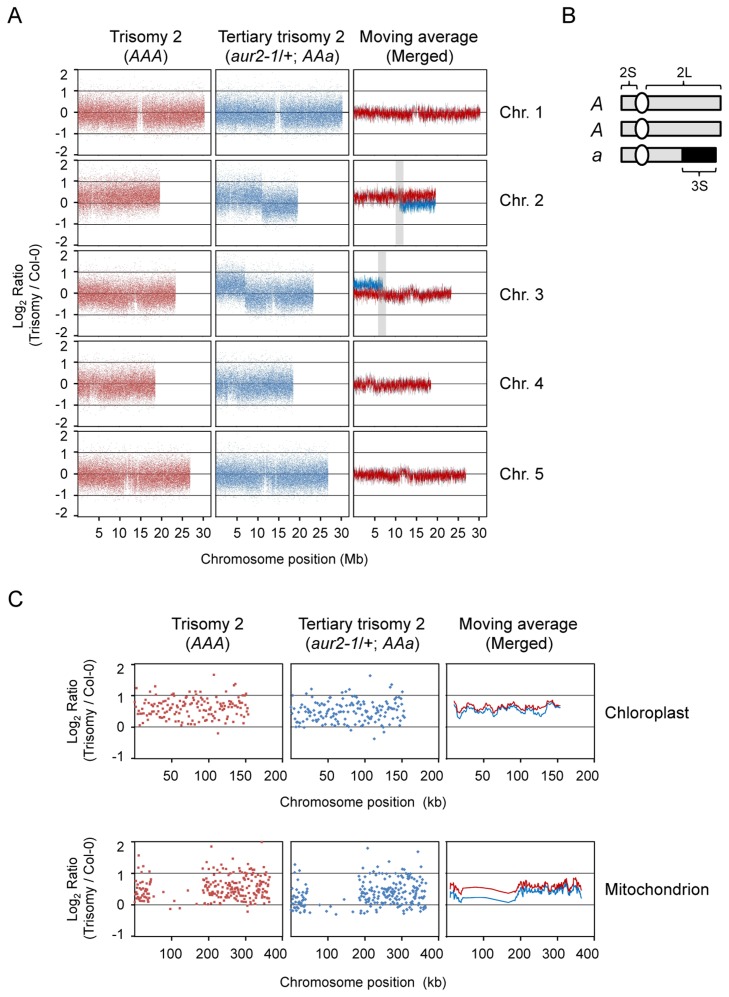
The *aur2-1*/+ is tertiary trisomy 2 revealed by array comparative genome hybridization (array CGH). (**A**) The log_2_ ratios of signal intensity of *Arabidopsis* chromosomes in trisomics (*AAA* or *AAa*) to wild-type diploids (Col-0). Each dot indicates one set of probes corresponding to a unique position on the chromosomes (Chr.). Locations of centromeres are the area with fewer dots. Shaded area in chromosome 2 indicates the region containing the translocation breakpoint. (**B**) A schematic representation of chromosome 2 in *aur2-1*/+. S, short; L, long arm of chromosomes. (**C**) The log_2_ ratios of signal intensity of chloroplast and mitochondria genomes in trisomy (*AAA* or *Aaa*) to wild-type diploid (Col-0). Genome duplication is identified by the ratio shifting above the baseline at 0. The moving average is a series of averages of 10 probe sets.

Two types of trisomy 2 progeny with round leaves were derived from self-fertilized *aur2-1*/+ plants: WT (*AAA*) and *aur2-1*/+ (*AAa*) (Fig. 1A, iii and iv). For simplicity, *A* and *a* hereafter represent chromosome 2 (Chr.2) carrying an *AUR2* and an *aur2-1* allele, respectively. To survey possible additional mutation(s) and validate an extra copy of Chr.2 in *aur2-1*/+, we used DNA tiling arrays for CGH (array CGH) to comprehensively inspect the whole genomes of WT trisomy (*AAA*), *aur2-1*/+ trisomy (*AAa*), and diploid Col-0 (*AA*) (Fig. 2A). In comparing the signal intensity of hybridized probes in Col-0 and the trisomic genomes (*AAA* and *AAa*), array CGH confirmed the chromosomal imbalance in the trisomic mutants. The ratio of probe intensities in *AAA* to Col-0 (red dots) indicates a duplication of Chr.2 in the *AAA* genome (Fig. 2A, left column). However, in the *aur2-1*/+ genome, only part of Chr.2 ( ˜11 Mb), including the whole short arm (2S) and part of the long arm (2L), showed increased signal intensity (Fig. 2A, blue dots). The signals of the remaining long arm of Chr.2 ( ˜8 Mb) were comparable to those for the WT genome (Fig. 2A). These results suggest that the extra Chr.2 in *aur2-1*/+ was broken in the middle of long arm and the region extending from the breakpoint was deleted. We found increased signals in the short arm of chromosome 3 (Chr.3S, ˜7 Mb), which indicates a chromosomal duplication in this region (Fig. 2A, middle column). Use of a moving average to merge two sets of signals revealed a simple duplication of Chr.2 in the *AAA* genome, with a deletion of Chr.2L and a duplication of Chr.3S in the *aur2-1*/+ genome. These results suggest a chromosomal translocation of the Chr.3S fragment to the Chr.2L region in the *aur2-1*/+ trisomic genome (Fig. 2B, 2n = 2x+T(2L/3S) = *AAa*). The findings are consistent with the cytogenetic data supporting that *aur2-1*/+ is tertiary trisomy 2. The extra chromosome could presumably be paired with the segments of the regular chromosome 2 and 3 to form a pentavalent at the diakinesis stage (Fig. 1C, viii-b).

We further determined the potential breakpoint in Chr.2L and Chr.3S (Fig. 2A, shaded regions) by plotting the ratio of hybridization signals against an 80-kb genomic region in the two trisomic genomes, *AAa* and *AAA* (Figure S2A in S1 File). The translocation breakpoint was mapped and plotted against the annotated genes. The *AUR2* locus (At2g25880) was located proximal to the Chr.2L breakpoint (Figure S2B in S1 File, upper panel) and the 3S breakpoint was at the 5′ end of At3g20040 (Figure S2B in S1 File, lower panel). The T-DNA insertion site of the *AUR2* locus was in close proximity to the breakpoint, which explains the tight association of *aur2-1* allele with the chromosomal translocation (Figure S2B in S1 File). The interchanged chromosome structure in the *aur2-1*/+ genome may have originated from a T-DNA insertion event.

Interestingly, we found increased intensity of the tiling probes representing genes in the chloroplast (plastid) and the mitochondrial genomes in both the tertiary (*aur2-1*/+) and primary (*AAA*) trisomy as compared with the diploid WT (Fig. 2C). Array CGH of *AAA* and *AAa* genomes revealed a comparable increase in chloroplast genomic sequences, which suggests the presence of homologous sequences in the trisomic genomes. Alternatively, the number of chloroplasts may be increased in the aneuploid cells. A 620-kb sequence proximal to the centromere region of Arabidopsis Chr.2 contains highly homologous sequences to the 350-kb mitochondrial genome [37], [38]. Thus, the increased mitochondrial sequences in the array CGH analysis of the two trisomics may result from the imbalanced copy number of Chr.2. However, a portion of the mitochondrion genome is not significantly enriched in the *AAa* genome, probably because of a translocation of the sub-chromosomal region of Chr.3 in the genome.

### Phenotypic consequences of chromosome translocation in *aur2-1*/+

**Figure 3:**
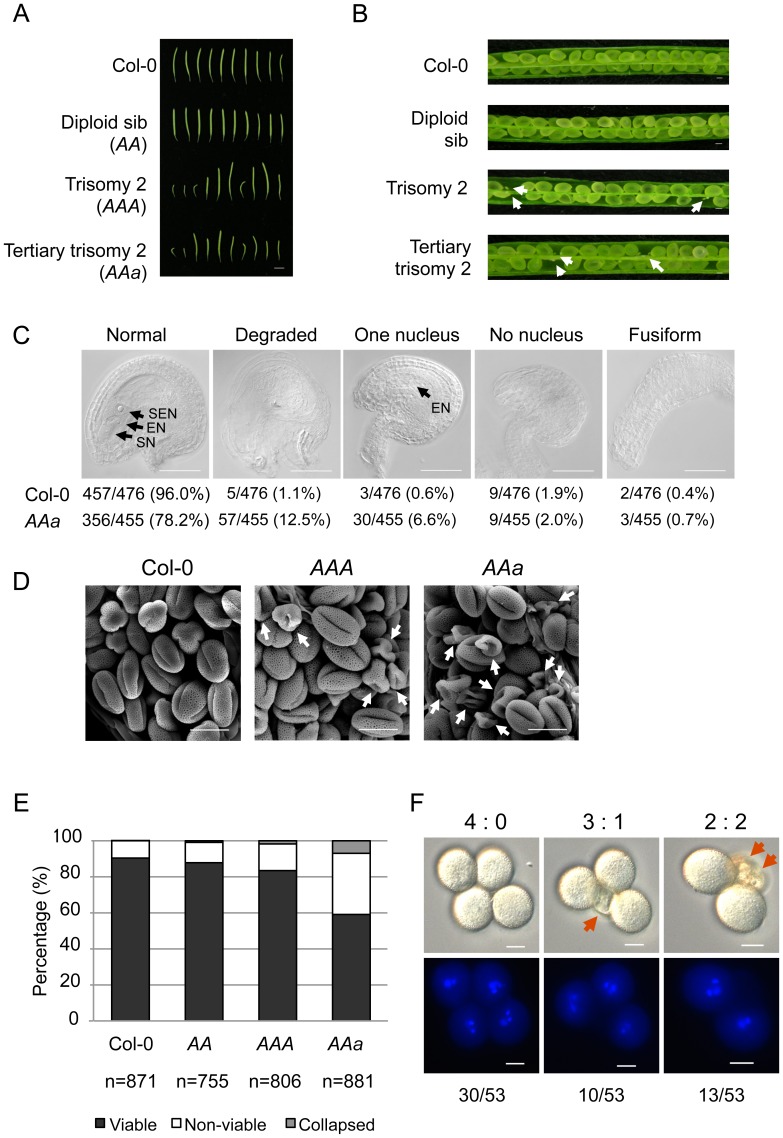
Phenotypic analyses in the trisomic plants. (**A**) Siliques from 7-week-old plants of wild type (Col-0), diploid sib (*AA*), trisomy 2 (*AAA*) and tertiary trisomy 2 (*aur2-1*/+; *AAa*) were collected and aligned from left to right in their order from bottom to top along the stems of individual plants. A similar phenotype was scored for at least 5 plants for each genetic background, and one representative set is shown. (**B**) Seed development in the siliques with comparable size shown in (**A**). Undeveloped seeds or aborted ovules are indicated by arrows. (**C**) Ovule development in Col-0 and *AAa* by DIC microscopy. Arrows indicate secondary endosperm nucleus (SEN), egg nucleus (EN) and synergid cell nucleus (SN). Quantitative data are indicated. (**D**) Morphology of pollen grains by scanning electron microscopy. Aberrant pollen grains are indicated by arrows. (**E**) Quantitative analysis of pollen viability by FDA staining. n, number of pollen grains scored. (**F**) The 4 products of 1 meiosis tetrad stay attached at the mature pollen stage in *aur2-1*/+ *qrt2-1*/*qrt2-1* double mutant. Representative DIC and fluorescence (DAPI) staining of the same mature pollen in upper and lower panels, respectively. Three types of segregation of normal pollen to aborted pollens (indicated by arrows) are shown above the upper panel and ratios from 53 tetrads are indicated below the lower panel. Scale bar in (**A**) 0.5 cm; (**B**) 200 µm; (**C**) 50 µm; (**D**) 20 µm; (**F**) 10 µm.

We showed a higher ratio of undeveloped seeds or unfertilized ovules in tertiary trisomy 2 (*AAa*, *aur2-1*/+) than WT plants (18.6% vs. 0.8%) (Table S1 and Figure S1C in S1 File). To determine which source of gametophytes, parental or maternal, was involved in the phenotype, we performed reciprocal crosses between *aur2-1*/+ and WT. Manually crossing *aur2-1*/+ with WT pollen (*aur2-1*/+ × Col-0) or ovules (Col-0×*aur2-1*/+) resulted in 14.7% and 8.3% undeveloped seeds, respectively, in F1 progeny (Table S1 in S1 File), which suggests that mutations in *aur2-1*/+ cause fertility defects in both parental and maternal gametophytes. To investigate whether the fertility phenotype was attributed to the trisomic genome or any additional mutations associated with *aur2-1*/+, we examined seed development in siliques of WT (Col-0), diploid sibs of WT (*AA*), trisomy 2 (*AAA*), and tertiary trisomy 2 (*AAa*) plants. We found undeveloped seeds and deformed siliques of irregular size along the stems in both *AAa* and *AAA* but not *AA* or WT plants, which suggests that the trisomic genome is mainly responsible for the phenotype (Figs. 3A and 3B).

Abnormal transmission of the *aur2-1* allele in self-fertilized *aur2-1*/+ may result from defective gametophytes, thus leading to sterility. To investigate this possibility, we examined the gametogenesis of ovules and pollen. We first inspected unfertilized ovules of *AAa* and WT by DIC light microscopy. The secondary endosperm nucleus (SEN) and egg nucleus (EN) in the embryo sac are clear, but in most cases, only 1 of the 2 synergid cell nuclei (SN) can be found in the same focal plane [39]. We found several types of abnormal ovules in both the WT and *AAa* plants (Fig. 3C). Degraded ovules still have the normal shape of ovules, but the boundaries of cells were unidentifiable (Fig. 3C, Degraded). Some of the ovules do not have an SEN and SN but have one EN (Fig. 3C, One nucleus), and some do not have any visible nucleus (Fig. 3C, No nucleus). A small number of ovules showed fusiform morphologic features (Fig. 3C, Fusiform). *AAa* plants appeared to have more “degraded” and “one-nucleus” ovules than the WT (Fig. 3C). Most of the WT ovules (96%) showed normal features as compared with *Aaa* (78.2%), so *aur2-1* is associated with defective ovule development. This observation agreed with findings that only 25.15% of the progeny carried the *aur2-1* allele transmitted from maternal gametophytes (Table 1).

We next examined the morphology and viability of pollen grains by Cryo-SEM and staining with FDA, respectively. *AAa* produced more aborted and shrunken pollen grains than did *AAA* and WT (Fig. 3D). Consistently, the proportion of viable pollen grains was comparable in WT and *AA* (approximately 90%) and slightly decreased in *AAA* (83.2%) but significantly reduced to 58.8% in *AAa* plants (Fig. 3E). Thus, although the trisomic genome (*AAA*) causes slightly reduced fertility, chromosome translocation associated with the *aur2-1* allele apparently results in a severe defect in pollen development. In addition to pollen development, the failed transmission of *aur2-1* allele by pollen might result from defects in pollen tube germination or elongation. The *in* *vitro* germination assay revealed more germinated pollen and elongated pollen tubes in the WT than in *AAa* (83.5% vs. 30.8%) (Figure S3A in S1 File). Therefore, both viability and germination of pollen grains were lower in *AAa* than the WT.

We introduced *quartet2* (*qrt2*) to generate the *aur2-1/*+ *qrt2-1*/*qrt2-1* double mutant for tetrad analysis. *QRT2* is essential for separating pollen grains from the 4 tetrads of meiosis during pollen development, so use of *qrt2* allows for analysis of allele segregation in *aur2-1*/+ pollen [40], [41]. We found 3 types of pollen tetrads in *aur2-1*/+ segregating at 4∶0 (30/53 = 56.6%), 3∶1 (10/53 = 18.9%), and 2∶2 (13/53 = 24.5%) viable to non-viable pollen (Fig. 3F, upper panel). Furthermore, DAPI staining of nuclear DNA revealed WT-like pollen grains in tetrads with 1 vegetative and 2 sperm nuclei, which were absent in degraded pollen grains (Fig. 3F, lower panel). Taken together, our results from genetic and morphological analyses of ovules and pollen indicate that both types of gametophytes in *aur2-1*/+ are defective to different degrees, which is likely responsible for the impaired transmission of *aur2-1* allele.

Additionally, we sought to determine whether embryo development was affected by the tertiary trisomy 2. The ovules from WT or *AAa* were manually fertilized with WT pollen and developing embryos were examined between 2 and 6 DAP. Most of the embryos (256/269 = 95.2%) were successfully developed from ovules of *AAa* fertilized with WT pollen as compared with embryos (242/243 = 99.6%) from fertilized WT ovules; however, the embryo development was slightly delayed for fertilized *AAa* ovules. For developing embryos at 6 DAP, 69.1% (186/269) and 25.7% (69/269) of fertilized ovules in *AAa* developed to the heart and torpedo stages, respectively, as compared with 37.9% (92/243) and 61.7% (150/243) of WT embryos, respectively (Figure S3B in S1 File). Therefore, the undeveloped seeds shown in Figure S1C in S1 File are not due to aborted embryo development but most likely to defective gametophytes for effective fertilization.

### Gene expression profiles in primary and tertiary trisomy 2

**Figure 4:**
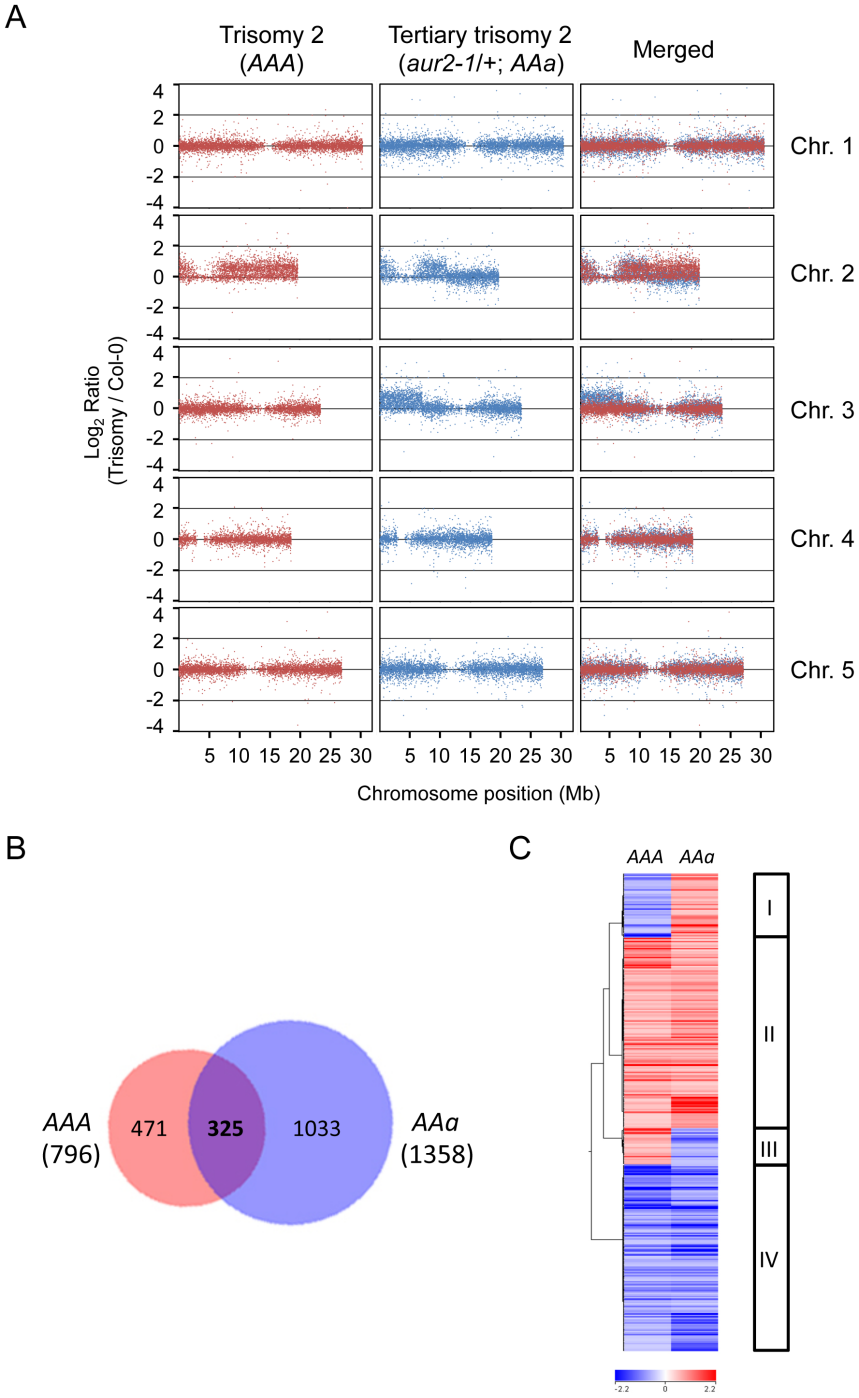
Whole-genome transcriptome analysis in trisomic plants. (**A**) The log_2_ ratios of the expression of all genes in trisomics (*AAA* or *AAa*) relative to wild-type diploids (Col-0). Each dot indicates one set of probes corresponding to a unique position on the chromosomes (Chr.). Positive and negative values indicate upregulated and downregulated expression, respectively. Locations of centromeres are the area with no gene expression. (**B**) Venn diagram shows the specific and overlapping genes with significant misregulated expression compared to wild-type diploid (Col-0) between trisomy 2 (*AAa*) and tertiary trisomy 2 (*AAA*) (*p*<0.05). (**C**) Clustering and heat map of the 325 misregulated genes common to the two trisomics (*AAA* and *AAa*). Four groups of genes based on expression patterns are shown on the right.

Phenotypic alterations resulting from genomic imbalance in aneuploidy involve changes in gene expression [1], [42]-[44]. To evaluate the global effect of the whole-chromosome and high-grade aneuploidy on gene expression, we used microarray assay to analyze the transcriptome profiles of WT, trisomy 2 (*AAA*), and tertiary trisomy 2 (*AAa*). Consistent with observations from array CGH (Fig. 2A), we found upregulated expression of genes in the triplicated chromosomes of the *AAA* and *AAa* corresponding to the trisomic chromosomal regions (Fig. 4A). We next investigated the expression patterns of genes on non-triplicated chromosomes [Chr.1, 3(3’), 4, 5] by normalization with the whole genome that excluded the trisomic regions as previously described [14]. To identify differentially expressed genes, we compared the gene expression profiles in the two trisomy 2 examples, *AAA* and *AAa*, with that in the WT (Fig. 4B, *p*<0.05). Interestingly, the number of misregulated genes in *AAa* (1358) was 1.7 times more than that in *AAA* (796), which indicates that the chromosomal translocation in the tertiary trisomy (*AAa*) affects more genes than the simple trisomy (*AAA*) (Fig. 4B). We further categorized 325 genes that were misregulated in both *AAA* and *AAa* into four groups based on the expression patterns and assigned GO terms to define functional properties (Fig. 4C). Approximately 78% of genes showed expression regulated by both *AAa* and *AAA* (Groups II and IV), whereas 22% were inversely regulated (Groups I and III).

GO term analysis revealed a significant enrichment of genes involved in defense response/incompatible interaction (false discovery rate [FDR] = 8.8E-24), immune response (FDR = 8.6E-09 and 2.1E-03), response to stimulus (FDR = 2E-23 and 5.7E-03) and stress (FDR = 1.0E-13 and 1.6E-04) (Table 3). Furthermore, GO terms related to response to chemical or hormone stimulus were enriched in both the upregulated (Group II, FDR = 3E-22 and 3.5E-15) and downregulated (Group IV, FDR = 2.5E-02 and 3.3E-02) groups of genes. Finally, genes involved in secondary metabolic processes (FDR = 1E-04) and lipid transport (FDR = 1.9E-09) were overrepresented as upregulated genes (Group II), whereas those responding to auxin stimulus (FDR = 3.5E-06) and carbohydrate metabolic process (FDR = 4.6E-02) were enriched as downregulated genes (Group IV). Our analysis suggests that cellular responses to different types of stimuli or stress may be evoked by both primary and tertiary trisomy 2, thus leading to physiological changes in regulating growth, hormone signaling and metabolic processes.

**Table 3:** Groups of enriched gene ontology (GO) terms for differentially expressed genes common to both trisomy 2 and tertiary trisomy 2.

Category	GO term no.	Description (Ontology^a^)	*p*-value	FDR^b^
Group I	GO:0009814	defense response, incompatible interaction (P)	1.4E-25	8.8E-24
	GO:0006955	immune response (P)	5.1E-10	8.6E-09
	GO:0051704	multi-organism process (P)	1.1E-06	1.0E-05
	GO:0006950	response to stress (P)	2.1E-05	1.6E-04
	GO:0050896	response to stimulus (P)	9.9E-04	5.7E-03
	GO:0016788	hydrolase activity, acting on ester bonds (F)	4.4E-03	4.5E-02
Group II	GO:0050896	response to stimulus (P)	1.8E-25	2.0E-23
	GO:0042221	response to chemical stimulus (P)	3.3E-24	3.0E-22
	GO:0009725	response to hormone stimulus (P)	7.0E-17	3.5E-15
	GO:0006950	response to stress (P)	2.5E-15	1.0E-13
	GO:0006869	lipid transport (P)	5.8E-11	1.9E-09
	GO:0006952	defense response (P)	8.6E-07	1.6E-05
	GO:0019748	secondary metabolic process (P)	5.9E-06	1.0E-04
	GO:0040007	growth (P)	5.5E-05	9.0E-04
	GO:0006955	immune response (P)	1.4E-04	2.1E-03
	GO:0003824	catalytic activity (F)	6.6E-04	3.1E-02
	GO:0003700	transcription factor activity (F)	2.1E-03	4.3E-02
Group III	GO:0007165	signal transduction (P)	3.3E-05	1.3E-03
	GO:0006350	transcription (F)	3.4E-03	3.4E-02
	GO:0005488	binding (F)	2.1E-03	3.8E-02
Group IV	GO:0009733	response to auxin stimulus (P)	1.1E-08	3.5E-06
	GO:0042221	response to chemical stimulus (P)	2.1E-04	2.5E-02
	GO:0009725	response to hormone stimulus (P)	5.3E-04	3.3E-02
	GO:0005975	carbohydrate metabolic process (P)	9.0E-04	4.6E-02

The gene ontology was analyzed by use of AgriGO [33].

a Biological process (P); molecular function (F).

b Chi-square statistical test; FDR, false discovery rate.

Because *AAa* is a tertiary trisomy 2 harboring a translocated chromosome 3 (Fig. 2B), we further examined the enriched GO terms present only in *AAA* or *AAa* (Table S2 in S1 File). In total, 471 and 1033 genes showed misregulated expression specifically in *AAA* and *AAa*, respectively (Fig. 4B). We found several GO cellular component terms related to organelles including plastid, mitochondrion, vacuole, endoplasmic reticulum, plasma membrane and cell wall were specifically enriched in *AAa* (Table S2 in S1 File). Furthermore, genes involved in several metabolic processes such as glycosinolate biosynthesis, sulfur metabolism, carbohydrate catabolism and metabolism were revealed among those misregulated specifically in *AAa*. Finally, GO terms related to biological process including cell cycle, cell death, proteolysis and chlorophyll metabolism were enriched only in *AAa*. The difference in specific gene sets misregulated in *AAA* and *AAa* likely results from the non-reciprocal translocation of Chr.3 to Chr.2 to generate the tertiary trisomy 2.

### Trans effect of trisomy 2 and 5 on gene expression profiles

**Figure 5:**
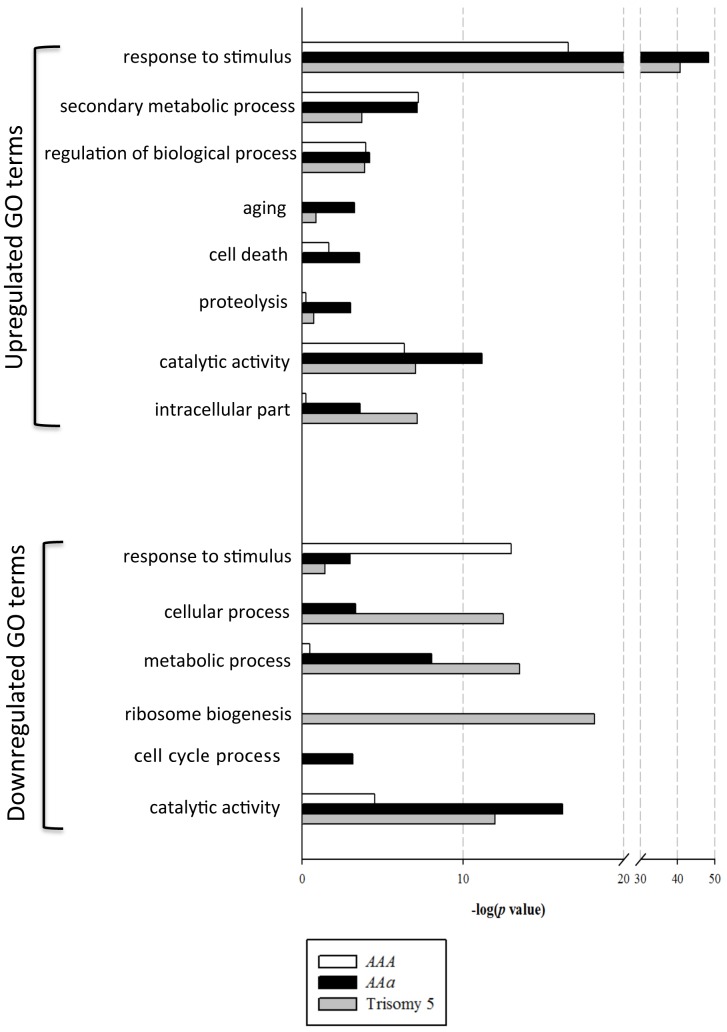
Gene ontology (GO) terms enriched among the misregulated genes in three trisomic plants. The specific enriched GO terms for up- and downregulated genes in *AAA* (white), *AAa* (black) and trisomy 5 (gray) were derived by use of agriGO [33]. The *p* values for GO terms are shown in –logarithmic scale. Data for trisomy 5 are from [5]. Complete lists are presented in Tables S3 and S4 in S1 File.

We next asked whether different trisomics would show a similar trans effect on changes of transcriptional signatures in Arabidopsis. By using the annotation enrichment tool (agriGO) we analyzed genes that were significantly misregulated (FDR<0.05) in the two trisomy 2 examples (*AAA* and *AAa*) and the trisomy 5, a primary trisomy with transcriptome data available [5]. Notably, we found GO terms related to response to stress and various types of endogenous and external stimuli and involved in catalytic activity were significantly enriched in both upregulated and downregulated groups of genes for all three trisomics (Fig. 5; Tables S3 and S4 in S1 File). Although several GO terms were commonly detected, misregulated genes in certain biological processes were specific to different trisomics. Genes involved in the flavonoid metabolic process and external encapsulating structure were upregulated and enriched only in trisomy 2 (*AAA and AAa*) but not trisomy 5 (Tables S3 and S4 in S1 File). Furthermore, genes related to transcription factor activity and DNA binding were upregulated and enriched only in primary trisomy 2 (*AAA*) and trisomy 5 but not tertiary trisomy 2 (*AAa*), while those involved in proteolysis, proteasome accessory complex, aging, cell death, cell cycle process, glucosinolate biosynthesis, carbohydrate metabolism and catabolism were specifically enriched in *AAa* (Fig. 5; Tables S3 and S4 in S1 File). Moreover, we found significant enrichment of several catalytic activity terms only in *AAa*, including that of oxidoreductases, transferases, and kinases (Table S4 in S1 File). In contrast, genes involved in nucleolus, ribosomal biogenesis, translation, ncRNA metabolism, several developmental processes, organelle and photosynthesis were overrepresented only in trisomy 5 (Fig. 5; Tables S3 and S4 in S1 File). Our observations agree with previous studies showing downregulation of genes related to cell growth, metabolic processes and proliferation in aneuploid cells, and upregulation of those associated with stress responses in eukaryotic organisms, including yeast, mice and humans [14], [45]-[47].

### Nuclear-encoded genes related to organelles are misregulated in the trisomics

Large-scale chromosomal changes in aneuploidy are proposed to affect the biogenesis and depletion of organelles that cause energy consumption, proteotoxic and aneuploidy-associated stresses [8], [15], [48]. The status of genomic unbalance urges cells to consume energy to overcome the detrimental effects of genetic abnormality in aneuploidy [20]. The plastids and mitochondria are the energy sources in plant cells [49]–[52] and the peroxisomes are essential organelles mediating many important metabolic pathways [53]. To understand the effect of trisomy on cellular energy and metabolism in Arabidopsis, we examined the expression profiles of nuclear-encoded genes related to plastids, mitochondria and peroxisomes [5]. In the non-triplicated WT chromosomes, 10.1%, 4.6%, and 2.1% of genes account for functions related to plastids, mitochondria and peroxisomes, respectively (Table 4, Wild type). However, in the non-triplicated chromosomes of trisomy 2, tertiary trisomy 2, and trisomy 5, the number of plastid-related genes increased to 12.4%, 14.6%, and 18.7%, respectively (Table 4). Additionally, mis-expressed genes related to mitochondria and peroxisomes were overrepresented in the genomes of tertiary trisomy 2 and trisomy 5, but not in trisomy 2 (Table 4). Consistent with the analysis of enriched GO terms, the number of differentially expressed genes related to organelles important for cellular energy and metabolism were significantly increased in at least two of the three trisomics examined, which suggests that aneuploid cells altered the expression of genes involved in these organelles to adapt to energy stress resulting from the imbalanced genome.

**Table 4:** The number of organelle-related genes that are significantly misregulated to different degrees in trisomic genomes.

	Gene number			
	Genome	Plastids^d^^,^ e	Mitochondria^d^^,^ e	Peroxisomes^d^^,^ e
	Genes on Chr.1, 3 (3’), 4, and 5^a^			
Wild-type diploid	14916	1513 (10.1%)	691 (4.6%)	313 (2.1%)
Trisomy 2^b^	1394	173^*^ (12.4%)	79 (5.7%)	38 (2.7%)
Tertiary trisomy 2^b^	1922	280^**^ (14.6%)	112^**^ (5.8%)	71^**^ (3.7%)
	**Genes on Chr.1, 2, 3, and 4** a			
Wild-type diploid	15030	1524 (10.1%)	710 (4.7%)	317 (2.1%)
Trisomy 5^c^	3584	669^**^ (18.7%)	222^**^ (6.2%)	125^**^ (3.5%)

a The probes matching multiple chromosomal locations are excluded.

b Misregulated genes (*p*<0.05) on Chr.1, 3 (3’ end; behind the translocation breakpoint), 4, and 5.

c Misregulated genes (*p*<0.05) on Chr.1, 2, 3, and 4; raw data from Huettel et al. (2008) [5].

d The genes related to plastids, mitochondria, and peroxisomes are from Law et al. (2012) [70].

e Chi-square test with Yates’ correction; two-tailed *P* value; ^*^, *p*<0.05; ^**^, *p*<0.01.

## Discussion

### Aneuploidy derived from T-DNA mutagenesis

Tertiary trisomy has been found in animals and plants and is considered to originate from the product of the malsegregation of interchange heterozygotes [2], [54]. Thus, the *aur2-1*/+ mutant may originate from a chromosome rearrangement that was likley induced by a T-DNA insertion event, followed by chromosome non-disjunction to become tertiary trisomy. However, with *aur2-1*/+, the malfunction of the T-DNA inserted gene may have increased the frequency of this phenomenon. AUR kinases play an important role in controlling the function of centrosomes, chromosome segregation and bipolar spindle assemble in yeast and mammalian cells [55]–[57]. Disruption in *AtAUR2* by the T-DNA insertion (*aur2-1*) may cause missegregation of chromosomes during gametophyte development, thus leading to trisomy. However, the reduced fertility in *aur2-1*/+ does not seem to directly associate with the *aur2-1* T-DNA insertion allele. We characterized another mutation in *AtAUR2* (*aur2-2*, GK403B02) and found that *aur2-2* was fertile in both male and female gametophytes, which is consistent with a previous report [21]. Therefore, the defect in transmission of *aur2-1* allele from male or female gametes is associated with *aur2-1*/+ trisomy but not *aur2-1 per se*.

The results from array CGH showed increased signal intensity in chloroplast and mitochondrial genomes for both *aur2-1*/+ (*AAa*) and primary trisomy 2 (*AAA*) (Fig. 2B). The homologous sequences of mitochondrion on the extra copy of Chr.2 may have enhanced the signal intensity of mitochondrial sequences in the trisomics on array CGH [37], [38]. Alternatively, the enhanced probe signals may indicate increased contents of both organelles in aneuploid cells. In fact, several types of aneuploidy in *Cyphomandra betacea* showed a higher copy number of chloroplasts than the diploid WT [58]. Recent reports indicated that aneuploid cells show increased need for energy that may help overcome the detrimental effects of overproduced proteins from the extra copies of genes [14], [20]. Both chloroplasts and mitochondria are the energy sources of plant cells; their maintenance and operation highly depend on proteins translated from nuclear-encoded genes [49]–[52]. Changes in large-scale gene expression in the aneuploidy may affect the replication and operation of these two organelles. This finding was supported by the gene content analysis in the Arabidopsis genome [38]. More than 10% of the annotated genes on Chr.2 are functionally associated with chloroplasts and mitochondria [38]. Disturbing the balance of these nuclear-encoded genes may also lead to changes in number of these two organelles.

### *AAa* genome is preferentially inherited in the offspring of tertiary trisomy 2

**Figure 6:**
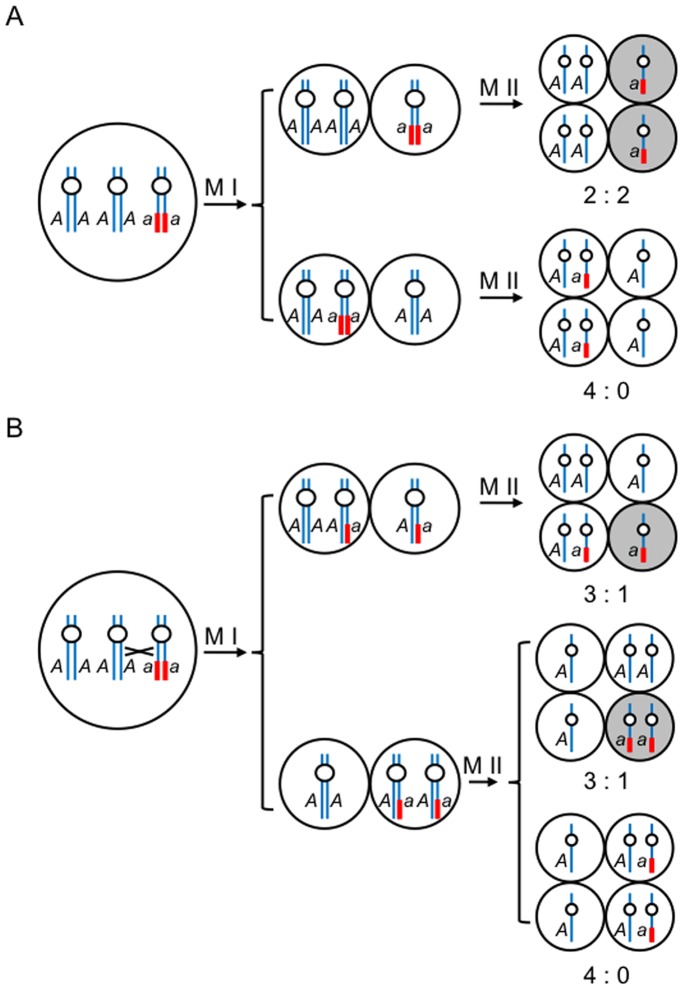
A Model of the genomic constitution of *aur2-1*/+ *qrt2-1*/*qrt2-1* tetrads. Meiosis without (**A**) or with crossing over (**B**). Left panel shows pollen mother cells and right panel, tetrads after meiosis. Chromosome 2 carrying the wild-type *AUR2* (*A*) is shown in a blue line or the *aur2-1* allele (*a*) with a translocation is indicated in a bold red line. M I, meiosis I; M II, meiosis II; shaded cells, aborted pollen grains.

Trisomy produces various progeny with different karyotypes and does not exclusively generate trisomic offspring [16]. The tertiary trisomy 2 (*AAa*) was intentionally and stably maintained in generations by selecting the *aur2-1* allele in this study. Because the *aur2-1* allele was present only in heterozygosity, the *aur2-1*/+ trisomy could be simplex (*Aaa*) or duplex (*AAa*). In the process of meiosis (M I and M II, Fig. 6), the haploid tetrads carrying *a* chromatids are expected to be degraded in subsequent microgametogenesis [59], [60]. The duplex genome (*AAa*) will show a ratio of 2∶2 or 4∶0 for viable to non-viable pollen following the rule of independent assortment (Fig. 6A), and 3∶1 or 4∶0 if a crossing-over takes place (Fig. 6B). In contrast, the simplex genome (*Aaa*) is expected to generate 2∶2 and 1∶3 patterns in pollen tetrads. Thus, our observation supports that *aur2-1*/+ is a duplex (*AAa*) (Fig. 3F).

An inheritance model by calculating the theoretical segregation ratio of progeny from self-fertilized *aur2-1*/+ (*AAa*) is shown in a Punnett square (Table S5 in S1 File). Among all possible combinations of progeny, the tetrasomics (*AAAA*, *AAAa*, and *AAaa*) would be rare because of the poor transmission of *n*+1 gametes [2], [16]. Reciprocal crosses showed no transmission of *Aa* or *a* from the male gametes (Table 1) and no *a* gametes from the female side (Table 2). Therefore, we found no trisomic (*Aaa*) or diploid (*Aa* and *aa*) progeny in the self-fertilized *aur2-1*/+ (Table S5 in S1 File, shaded boxes). Thus, 3 major types of progeny would be generated – *AAA*, *AAa* and *AA* (Table S5 in S1 File, white boxes). Theoretically, the number of trisomic progeny carrying genotypes *AAA* and *AAa* should be equivalent (*AAA*:*AAa* = (2/36+2/36): 4/36 = 1∶1). Because the male *n*+1 gametes (*AA*) were rarely transmitted [16], the expected ratio between the *AAA* and *AAa* offspring would be skewed and close to 1∶2 (*AAA*:*AAa* = 2/36∶4/36 = 1∶2). Surprisingly, we found 11∶72 and 4∶26 ratios of *AAA*:*AAa* in the progeny of self-fertilized *aur2-1*/+ and *aur2-1*/+ ovules crossed with WT pollen, respectively (Table 2); therefore, the *AAa* genome is preferentially inherited in the offspring of *aur2-1*/+. The preferential inheritance of the T-DNA insertion (*aur2-1*) may be due to its translocated chromosomal structure (Fig. 2B), which contains portions of Chr.2 and Chr.3, and will not be a perfect pairing partner to either one. The WT chromosomes may pair in higher frequency and ensure that each copy moves to the opposite poles in first division. If this is the case, the T-DNA carrying chromosome (*a*) is rarely alone and expected to accompanied by a normal chromosome (*A*) in one side [2].

Our findings suggest that the unique genomic structure of the tertiary trisomy 2 likely provided a selective advantage among progeny. Poor transmission of the T-DNA–inserted chromosome (*a*) in both male and female gametes resulted in limited genotypes in the offspring of self-fertilized *aur2-1*/+ (Table S5 in S1 File). Preferential inheritance from female gametes led to better survival of the T-DNA–carrying progeny (*AAa*) than the other trisomic offspring (*AAA*). Chromosome translocation is frequently associated with T-DNA insertion mutagenesis, about 19% [18]. Similar cases may show preferential inheritance in other chromosomal rearrangements resulting from T-DNA insertion. Chromosome non-disjunction may arise occasionally and thus led to tertiary trisomy. Further studies to investigate the occurrence of aneuploidy in the T-DNA insertion mutant library will be required to test this possibility.

### Transcriptional consequences of aneuploidy in Arabidopsis

By using expression microarray analysis, we found a trans effect of triplicated genes on the diploid chromosomes to reveal genes involved in stress response, organelle-related functions, and transcription factors were misregulated and overrepresented in the trisomy (Fig. 5; Tables S2–S4 in S1 File). Our observations are consistent with previous reports of aneuploidy in plants and other organisms [5], [14], [42], [61]. Our analyses of the global gene expression profiles support a hypothesis that, regardless the type of trisomy, genes involved in stress response, defense response, energy and metabolism processes are significantly affected by aneuploid genomes. Previous studies showed increased transcription of environmental stress response (ESR)-like genes in all types of aneuploid cells in yeast [14], [62], plants [5], [61], mice and humans [14], [63]. The trans effect on gene expression may be attributed to regulatory proteins encoded by some of the triplicated genes to modulate concurrently on overlapping targets. Alternatively, the differential gene expression in aneuploid cells is a consequence, rather than the cause, of transcription failure.

Several specific GO terms were identified for misregulated genes in different trisomics. For example, the genes related to transcription were overrepresented in primary trisomics (trisomy 2 and trisomy 5) but not tertiary trisomy 2. In contrast, genes related to proteolysis, aging and cell death were significantly enriched in tertiary trisomy 2 but not the other primary trisomics. Furthermore, tertiary trisomy 2 seemed to show specific involvement in regulation of cell cycle regulation and carbohydrate metabolism. Mouse models of Down syndrome, Ts65Dn and Ts1Cje, show a connection between the high-grade aneuploidy and cellular alterations in protein degradation and aging [64]. The Ts65Dn mouse is a tertiary trisomy containing the distal region of mouse Chr.16 (92 genes homologous to nearly two-thirds of the human Chr.21) and Chr.17, whereas the Ts1Cje mouse carries a smaller region of mouse Chr.16 (67 genes homologous to the human Chr.21) and telomeric region of Chr.12. Both mouse models showed a combinational effect on phenotypes involving impaired protein homeostasis phenotype, such as cognitive abnormalities, age-related atrophy, degeneration of cholinergic neurons, age-related endosomal pathologies and protein misfolding [64]. Aneuploid cells may require more time to properly align chromosomes at metaphase. As a consequence of having extra chromosomes, tumor cells would be more prone to chromosome missegregation and aberrant in cell cycle progression because of checkpoint abrogation (reviewed in [48], [65]). Thus, the high-grade aneuploidy may affect additional genes located on other chromosomes, which combined with a gene dosage effect, influences gene expression patterns. Whether the genomic instability evoked by aneuploidy contributes to the affected plant phenotypes or such transcriptional changes are the result of differential growth fitness remains to be tested.

Taken together, the tertiary trisomy *aur2-1*/+ (*AAa*) has multiple traits: round leaves, aborted seeds, underdeveloped siliques with irregular size, defective ovule development, poor pollen viability, germination and pollen tube elongation, and abnormal pollen morphology. These phenotypes appeared to be caused by several factors. First, the most prominent round leaf phenotype was attributed to the effect of chromosomal imbalance by an extra Chr.2 [16]. In *AAa*, the extra chromosome contained ˜7 Mb chromosome 3S and the whole short arm 2S recombined with half of the long arm 2L (Fig. 2B and Figure S2 in S1 File). Large chromosomal deletion combined with translocation should affect more genes in *AAa* than *AAA* [66]. This possibility is supported by a previous report that the longer chromosomal component in the trisomic chromosome provided more profound morphological effect than the short one in tomato [67]. Second, the poor pollen viability and defective male gametophyte development may be caused by the translocated structure of the extra chromosome in *aur2-1*. Tertiary trisomy resulting in low pollen fertility was found in pearl millet ( ˜7%) [68] and lentil ( ˜9%) [69]. In mammals, most trisomy causes adverse effects on embryo development and is fatal to fetuses, leading to spontaneous abortion [8]. Finally, gene expression profiling provided useful information to compare and contrast the effects of different degrees of aneuploidy on plant cell physiology.

## Supporting Information

S1 File **Figure S1, Seed development in self-fertilized and manually crossed** ***aur2-1*** **/+.** (**A**) Schematic representation of T-DNA insertion site in the *AtAUR2* locus. The upstream region is shown as a line and exons as black boxes. The locations of primers (LP and RP) flanking *AtAUR2* and T-DNA–specific primer (BP) are indicated by arrows. (**B**) Genotyping of wild type (Col-0) and *aur2-1*/+. G, PCR product amplified by LP and RP primers for wild type allele; T, amplification by BP and RP primers for T-DNA allele. (**C**) Seed development in the siliques of the self-fertilized wild type (Col-0), *aur2-1*/+ and F1 siliques from reciprocal crosses (female×male). Arrows indicate undeveloped seeds. Scale bar, 200 µm. **Figure S2, Translocation breakpoints of the tertiary trisomy 2 (** ***aur2-1*** **/+;** ***AAa*** **).** (**A**) A zoom-in diagram from the shaded region of the chromosome 2 in the Figure 2A. The translocation breakpoint is mapped within the shaded region in pink. The moving average of the ratio of signal intensities in 2 trisomy genomes is shown. (**B**) Locations of the *AUR2* locus (At2g25880) with a T-DNA insertion (inverted triangle) and adjacent annotated genes within the shaded region in (**A**) (upper panel). Locations of annotated genes and translocation breakpoint (arrow) on the chromosome 3 (lower panel). **Figure S3, Pollen germination and embryo development in tertiary trisomy 2.** (**A**) Pollen grains from *aur2-1*/+ show impaired pollen germination and pollen tube growth. The *in* *vitro* pollen germination of pollen grains from the wild type (Col-0) and *aur2-1*/+ were scored by microscopy and measured by use of ImageJ after 8-h incubation in germination agar medium. Data are mean±SD from 3 independent experiments (n = 400 ˜500). (**B**) Representative differential interference contrast (DIC) images of developing embryos in wild type (Col-0) and tertiary trisomy 2 (*aur2-1*/+; *AAa*) whose ovules were both manually pollinated by WT pollen. Quantitative data of the embryos at 6 days after pollination (DAP) are shown. Scale bar, 100 µm. **Figure S4,** ***M(A)*** **plots of the intensity differences between the trisomics and WT.** (**A**) *M(A)* plot of trisomy 2; (**B**) *M(A)* plot of tertiary trisomy 2. The *M* values (log_2_ ratios) of the trisomy 2 and wild type (Col-0) are represented in the *y-*axis and the *A* values (the average expression) between the trisomics are represented in the *x-*axis. Red and blue dots, entities of the triplicated chromosomes; black dots, entities of other non-triplicated chromosomes; purple and orange lines, moving average. Upper and lower lines of the purple or orange lines, moving average ± SD. Sufficiently strongly expressed transcripts (the dots to the right of the dash lines) were isolated to perform the transcriptional signature analysis in this study. The methodology of data analysis is from Huettel et al. (2008) [5]. **Table S1, The ratio of undeveloped^ seeds^ in the progeny of self-fertilized and manual crosses of** ***aur2-1*** **/+ and wild-type (Col-0) plants. Table S2, Enriched gene ontology (GO) terms of genes that are misregulated specifically in the trisomy 2 or tertiary trisomy 2. Table S3, Enriched GO terms of significantly upregulated genes in the trisomy 2, tertiary trisomy 2, and trisomy 5. Table S4, Enriched GO terms of significantly downregulated genes in the trisomy 2, tertiary trisomy 2, and trisomy 5.**
(PDF)

## References

[1] Henry IM, Dilkes BP, Miller ES, Burkart-Waco D, Comai L (2010) Phenotypic consequences of aneuploidy in *Arabidopsis thaliana* . Genetics 186:1231–1245. 20876566 10.1534/genetics.110.121079 PMC2998307

[2] Singh RJ (1993) Plant cytogenetics. Boca Raton: CRC Press. 391 p.

[3] Hassold T, Hunt P (2001) To err (meiotically) is human: The genesis of human aneuploidy. Nat Rev Genet 2:280–291. 11283700 10.1038/35066065

[4] Megarbane A, Ravel A, Mircher C, Sturtz F, Grattau Y, et al (2009) The 50th anniversary of the discovery of trisomy 21: the past, present, and future of research and treatment of Down syndrome. Genet Med 11:611–616. 19636252 10.1097/GIM.0b013e3181b2e34c

[5] Huettel B, Kreil DP, Matzke M, Matzke AJM (2008) Effects of aneuploidy on genome structure, expression, and interphase organization in *Arabidopsis thaliana* . PLoS Genet 4:e1000226. 18927630 10.1371/journal.pgen.1000226 PMC2562519

[6] Matzke MA, Moscone EA, Park YD, Papp I, Oberkofler H, et al (1994) Inheritance and expression of a transgene insert in an aneuploid tobacco line. Mol Gen Genet 245:471–485. 7808397 10.1007/BF00302260

[7] Papp I, Iglesias VA, Moscone EA, Michalowski S, Spiker S, et al (1996) Structural instability of a transgene locus in tobacco is associated with aneuploidy. Plant J 10:469–478. 8811861 10.1046/j.1365-313x.1996.10030469.x

[8] Siegel JJ, Amon A (2012) New insights into the troubles of aneuploidy. Annu Rev Cell Dev Biol 28:189–214. 22804579 10.1146/annurev-cellbio-101011-155807 PMC3919630

[9] Gordon DJ, Resio B, Pellman D (2012) Causes and consequences of aneuploidy in cancer. Nat Rev Genet 13:189–203. 22269907 10.1038/nrg3123

[10] Storchova Z (2012) The Causes and Consequences of Aneuploidy in Eukaryotic Cells. In: Storchova Z, editor. Aneuploidy in Health and Disease: InTech.

[11] McGranahan N, Burrell RA, Endesfelder D, Novelli MR, Swanton C (2012) Cancer chromosomal instability: therapeutic and diagnostic challenges. EMBO Rep 13:528–538. 22595889 10.1038/embor.2012.61 PMC3367245

[12] Matzke MA, Mette MF, Kanno T, Matzke AJM (2003) Does the intrinsic instability of aneuploid genomes have a causal role in cancer? Trends Genet 19:253–256. 12711216 10.1016/s0168-9525(03)00057-x

[13] Sheltzer JM, Amon A (2011) The aneuploidy paradox: costs and benefits of an incorrect karyotype. Trends Genet 27:446–453. 21872963 10.1016/j.tig.2011.07.003 PMC3197822

[14] Sheltzer JM, Torres EM, Dunham MJ, Amon A (2012) Transcriptional consequences of aneuploidy. Proc Natl Acad Sci USA 109:12644–12649. 22802626 10.1073/pnas.1209227109 PMC3411958

[15] Pfau SJ, Amon A (2012) Chromosomal instability and aneuploidy in cancer: from yeast to man. EMBO Rep 13:515–527. 22614003 10.1038/embor.2012.65 PMC3367249

[16] Koornneef M, der Veen JHV (1983) Trisomics in *Arabidopsis thaliana* and the location of linkage groups. Genetica 61:41–46.

[17] O’Malley RC, Ecker JR (2010) Linking genotype to phenotype using the Arabidopsis unimutant collection. Plant J 61:928–940. 20409268 10.1111/j.1365-313X.2010.04119.x

[18] Clark KA, Krysan PJ (2010) Chromosomal translocations are a common phenomenon in *Arabidopsis thaliana* T-DNA insertion lines. Plant J 64:990–1001. 21143679 10.1111/j.1365-313X.2010.04386.x PMC3079379

[19] Ohba T, Yoshioka Y, Machida C, Machida Y (1995) DNA rearrangement associated with the integration of T-DNA in tobacco: an example for multiple duplications of DNA around the integration target. Plant J 7:157–164. 7894506 10.1046/j.1365-313x.1995.07010157.x

[20] Tang YC, Amon A (2013) Gene copy-number alterations: a cost-benefit analysis. Cell 152:394–405. 23374337 10.1016/j.cell.2012.11.043 PMC3641674

[21] Van Damme D, De Rybel B, Gudesblat G, Demidov D, Grunewald W, et al (2011) *Arabidopsis* alpha Aurora kinases function in formative cell division plane orientation. Plant Cell 23:4013–4024. 22045917 10.1105/tpc.111.089565 PMC3246319

[22] Heslop-Harrison J, Heslop-Harrison Y (1970) Evaluation of pollen viability by enzymatically induced fluorescence; intracellular hydrolysis of fluorescein diacetate. Stain Technol 45:115–120. 4192549 10.3109/10520297009085351

[23] Fan LM, Wang YF, Wang H, Wu WH (2001) In vitro Arabidopsis pollen germination and characterization of the inward potassium currents in Arabidopsis pollen grain protoplasts. J Exp Bot 52:1603–1614. 11479325

[24] Park SK, Howden R, Twell D (1998) The *Arabidopsis thaliana* gametophytic mutation *gemini pollen1* disrupts microspore polarity, division asymmetry and pollen cell fate. Development 125:3789–3799. 9729487 10.1242/dev.125.19.3789

[25] Alvarez-Buylla ER, Benítez M, Corvera-Poiré A, Chaos Cador Á, de Folter S, et al. (2010) Flower development. The Arabidopsis Book: e0127. 10.1199/tab.0127 PMC3244948 22303253

[26] Boisnard-Lorig C, Colon-Carmona A, Bauch M, Hodge S, Doerner P, et al (2001) Dynamic analyses of the expression of the HISTONE::YFP fusion protein in Arabidopsis show that syncytial endosperm is divided in mitotic domains. Plant Cell 13:495–509. 11251092 10.1105/tpc.13.3.495 PMC135513

[27] Ross KJ, Fransz P, Jones GH (1996) A light microscopic atlas of meiosis in *Arabidopsis thaliana* . Chromosome Res 4:507–516. 8939362 10.1007/BF02261778

[28] Chang YC, Shii CT, Chung MC (2009) Variations in ribosomal RNA gene loci in spider lily (*Lycoris* spp.). J Am Soc Hortic Sci 134:567–573.

[29] Chung MC, Lee YI, Cheng YY, Chou YJ, Lu CF (2008) Chromosomal polymorphism of ribosomal genes in the genus Oryza. Theor Appl Genet 116:745–753. 18214422 10.1007/s00122-007-0705-z PMC2271086

[30] Kent WJ (2002) BLAT - The BLAST-like alignment tool. Genome Res 12:656–664. 11932250 10.1101/gr.229202 PMC187518

[31] Wu Z, Irizarry RA, Gentleman R, Martinez-Murillo F, Spencer F (2004) A Model-Based Background Adjustment for Oligonucleotide Expression Arrays. Journal of the American Statistical Association 99:909–917.

[32] Huber W, von Heydebreck A, Sültmann H, Poustka A, Vingron M (2002) Variance stabilization applied to microarray data calibration and to the quantification of differential expression. Bioinformatics 18:S96–S104. 12169536 10.1093/bioinformatics/18.suppl_1.s96

[33] Du Z, Zhou X, Ling Y, Zhang Z, Su Z (2010) agriGO: a GO analysis toolkit for the agricultural community. Nucleic Acids Res 38:W64–70. 20435677 10.1093/nar/gkq310 PMC2896167

[34] Sears LMS, Lee-Chen S (1970) Cytogenetic studies in *Arabidopsis thaliana* . Can J Genet Cytol 12:217–223.

[35] Koornneef M, Fransz P, de Jong H (2003) Cytogenetic tools for *Arabidopsis thaliana* . Chromosome Res 11:183–194. 12769286 10.1023/a:1022827624082

[36] Kosterin OE, Galieva ER, Bogdanova VS (2009) The first record of tetrasomy in pea (*Pisum sativum* L.). Euphytica 166:109–121.

[37] Stupar RM, Lilly JW, Town CD, Cheng Z, Kaul S, et al (2001) Complex mtDNA constitutes an approximate 620-kb insertion on Arabidopsis thaliana chromosome 2: implication of potential sequencing errors caused by large-unit repeats. Proceedings of the National Academy of Sciences of the United States of America 98:5099–5103. 11309509 10.1073/pnas.091110398 PMC33170

[38] Lin X, Kaul S, Rounsley S, Shea TP, Benito MI, et al (1999) Sequence and analysis of chromosome 2 of the plant *Arabidopsis thaliana* . Nature 402:761–768. 10617197 10.1038/45471

[39] Drews GN, Lee D, Christensen CA (1998) Genetic analysis of female gametophyte development and function. Plant Cell 10:5–17. 9477569 10.1105/tpc.10.1.5 PMC143932

[40] Preuss D, Rhee SY, Davis RW (1994) Tetrad analysis possible in *Arabidopsis* with mutation of the *QUARTET* (*QRT*) genes. Science 264:1458–1460. 8197459 10.1126/science.8197459

[41] Copenhaver GP, Keith KC, Preuss D (2000) Tetrad analysis in higher plants. A budding technology. Plant Physiol 124:7–15. 10982416 10.1104/pp.124.1.7 PMC1539273

[42] Birchler JA, Veitia RA (2007) The gene balance hypothesis: from classical genetics to modern genomics. Plant Cell 19:395–402. 17293565 10.1105/tpc.106.049338 PMC1867330

[43] Birchler JA, Veitia RA (2010) The gene balance hypothesis: implications for gene regulation, quantitative traits and evolution. New Phytol 186:54–62. 19925558 10.1111/j.1469-8137.2009.03087.x PMC2858765

[44] Birchler JA, Veitia RA (2014) The Gene Balance Hypothesis: dosage effects in plants. Methods Mol Biol 1112:25–32. 24478005 10.1007/978-1-62703-773-0_2

[45] Sheltzer JM (2013) A transcriptional and metabolic signature of primary aneuploidy is present in chromosomally unstable cancer cells and informs clinical prognosis. Cancer Res 73:6401–6412. 24041940 10.1158/0008-5472.CAN-13-0749 PMC3901577

[46] Sheltzer JM, Blank HM, Pfau SJ, Tange Y, George BM, et al (2011) Aneuploidy drives genomic instability in yeast. Science 333:1026–1030. 21852501 10.1126/science.1206412 PMC3278960

[47] Williams BR, Prabhu VR, Hunter KE, Glazier CM, Whittaker CA, et al (2008) Aneuploidy affects proliferation and spontaneous immortalization in mammalian cells. Science 322:703–709. 18974345 10.1126/science.1160058 PMC2701511

[48] Oromendia AB, Amon A (2014) Aneuploidy: implications for protein homeostasis and disease. Dis Model Mech 7:15–20. 24396150 10.1242/dmm.013391 PMC3882044

[49] Mackenzie S, McIntosh L (1999) Higher plant mitochondria. Plant Cell 11:571–585. 10213779 10.1105/tpc.11.4.571 PMC144202

[50] Pogson BJ, Woo NS, Forster B, Small ID (2008) Plastid signalling to the nucleus and beyond. Trends Plant Sci 13:602–609. 18838332 10.1016/j.tplants.2008.08.008

[51] Liere K, Weihe A, Borner T (2011) The transcription machineries of plant mitochondria and chloroplasts: Composition, function, and regulation. J Plant Physiol 168:1345–1360. 21316793 10.1016/j.jplph.2011.01.005

[52] Eberhard S, Drapier D, Wollman FA (2002) Searching limiting steps in the expression of chloroplast-encoded proteins: relations between gene copy number, transcription, transcript abundance and translation rate in the chloroplast of *Chlamydomonas reinhardtii* . Plant J 31:149–160. 12121445 10.1046/j.1365-313x.2002.01340.x

[53] Zhang X, Shiu SH, Cal A, Borevitz JO (2008) Global analysis of genetic, epigenetic and transcriptional polymorphisms in *Arabidopsis thaliana* using whole genome tiling arrays. PLoS Genet 4:e1000032. 18369451 10.1371/journal.pgen.1000032 PMC2265482

[54] Braddock SR, Henley KM, Potter KL, Nguyen HG, Huang THM (2000) Tertiary trisomy due to a reciprocal translocation of chromosomes 5 and 21 in a four-generation family. Am J Med Genet 92:311–317. 10861659 10.1002/1096-8628(20000619)92:5<311::aid-ajmg4>3.0.co;2-7

[55] Chan CSM, Botstein D (1993) Isolation and characterization of chromosome-gain and increase-in-ploidy mutants in yeast. Genetics 135:677–691. 8293973 10.1093/genetics/135.3.677 PMC1205712

[56] Bischoff JR, Anderson L, Zhu YF, Mossie K, Ng L, et al (1998) A homologue of *Drosophila aurora* kinase is oncogenic and amplified in human colorectal cancers. EMBO J 17:3052–3065. 9606188 10.1093/emboj/17.11.3052 PMC1170645

[57] Fu JY, Bian ML, Jiang Q, Zhang CM (2007) Roles of aurora kinases in mitosis and tumorigenesis. Mol Cancer Res 5:1–10. 17259342 10.1158/1541-7786.MCR-06-0208

[58] Standring LS, Pringle GJ, Murray BG (1990) The control of chloroplast number in *Solanum muricatum* Ait. and *Cyphomandra betacea* (Cav.) Sendt. and its value as an indicator of polyploidy. Euphytica 47:71–77.

[59] Chen YCS, McCormick S (1996) Sidecar pollen, an *Arabidopsis thaliana* male gametophytic mutant with aberrant cell divisions during pollen development. Development 122:3243–3253. 8898236 10.1242/dev.122.10.3243

[60] Cai XW, Xu SS (2007) Meiosis-driven genome variation in plants. Curr Genomics 8:151–161. 18645601 10.2174/138920207780833847 PMC2435351

[61] Makarevitch I, Harris C (2010) Aneuploidy causes tissue-specific qualitative changes in global gene expression patterns in maize. Plant Physiol 152:927–938. 20018594 10.1104/pp.109.150466 PMC2815861

[62] Torres EM, Sokolsky T, Tucker CM, Chan LY, Boselli M, et al (2007) Effects of aneuploidy on cellular physiology and cell division in haploid yeast. Science 317:916–924. 17702937 10.1126/science.1142210

[63] Oromendia AB, Dodgson SE, Amon A (2012) Aneuploidy causes proteotoxic stress in yeast. Genes Dev 26:2696–2708. 23222101 10.1101/gad.207407.112 PMC3533075

[64] Cataldo AM, Petanceska S, Peterhoff CM, Terio NB, Epstein CJ, et al (2003) App gene dosage modulates endosomal abnormalities of Alzheimer’s disease in a segmental trisomy 16 mouse model of down syndrome. J Neurosci 23:6788–6792. 12890772 10.1523/JNEUROSCI.23-17-06788.2003 PMC6740714

[65] Manchado E, Guillamot M, Malumbres M (2012) Killing cells by targeting mitosis. Cell Death Differ 19:369–377. 22223105 10.1038/cdd.2011.197 PMC3278741

[66] Vizir IY, Mulligan BJ (1999) Genetics of gamma-irradiation-induced mutations in *Arabidopsis thaliana*: large chromosomal deletions can be rescued through the fertilization of diploid eggs. J Hered 90:412–417. 10355125 10.1093/jhered/90.3.412

[67] Khush GS, Rick CM (1967) Tomato tertiary trisomics - origin, identification, morphology and use in determining poiition of centromeres and arm location of markers. Can J Genet Cytol 9:610–631.

[68] Kumar RS, Singh UP, Singh RM, Singh RB (1985) Tertiary trisomics and their use in pearl millet improvement. Cytologia 50:433–443.

[69] Ladizinsky G, Weeden NF, Muehlbauer FJ (1990) Tertiary trisomics in lentil. Euphytica 51:179–184.

[70] Law SR, Narsai R, Taylor NL, Delannoy E, Carrie C, et al (2012) Nucleotide and RNA metabolism prime translational initiation in the earliest events of mitochondrial biogenesis during Arabidopsis germination. Plant Physiol 158:1610–1627. 22345507 10.1104/pp.111.192351 PMC3320173

