# Supplementary material for: Transcriptional Consequence and Impaired Gametogenesis with High-Grade Aneuploidy in Arabidopsis thaliana
Source: PLoS One. 2014 Dec 16;9(12):e114617. doi: 10.1371/journal.pone.0114617 (PMC4267805; doi:10.1371/journal.pone.0114617)
Supplement: S1 File — Figure S1, Seed development in self-fertilized and manually crossed aur2-1 /+. (A) Schematic representation of T-DNA insertion site in the AtAUR2 locus. The upstream region is shown as a line and exons as black boxes. The locations of primers (LP and RP) flanking AtAUR2 and T-DNA–specific primer (BP) are indicated by arrows. (B) Genotyping of wild type (Col-0) and aur2-1/+. G, PCR product amplified by LP and RP primers for wild type allele; T, amplification by BP and RP primers for T-DNA allele. (C) Seed development in the siliques of the self-fertilized wild type (Col-0), aur2-1/+ and F1 siliques from reciprocal crosses (female×male). Arrows indicate undeveloped seeds. Scale bar, 200 µm. Figure S2, Translocation breakpoints of the tertiary trisomy 2 ( aur2-1 /+; AAa ). (A) A zoom-in diagram from the shaded region of the chromosome 2 in the Figure 2A. The translocation breakpoint is mapped within the shaded region in pink. The moving average of the ratio of signal intensities in 2 trisomy genomes is shown. (B) Locations of the AUR2 locus (At2g25880) with a T-DNA insertion (inverted triangle) and adjacent annotated genes within the shaded region in (A) (upper panel). Locations of annotated genes and translocation breakpoint (arrow) on the chromosome 3 (lower panel). Figure S3, Pollen germination and embryo development in tertiary trisomy 2. (A) Pollen grains from aur2-1/+ show impaired pollen germination and pollen tube growth. The in vitro pollen germination of pollen grains from the wild type (Col-0) and aur2-1/+ were scored by microscopy and measured by use of ImageJ after 8-h incubation in germination agar medium. Data are mean±SD from 3 independent experiments (n = 400 ˜500). (B) Representative differential interference contrast (DIC) images of developing embryos in wild type (Col-0) and tertiary trisomy 2 (aur2-1/+; AAa) whose ovules were both manually pollinated by WT pollen. Quantitative data of the embryos at 6 days after pollination (DAP) are shown. Scale b [file pone.0114617.s001.pdf]

Supporting Information

Transcriptional consequence and impaired gametogenesis with high-grade aneuploidy in *Arabidopsis thaliana*

Kuan-Lin Lo[1,§], Long-Chi Wang[2,§], I-Ju Chen[1], Yu-Chen Liu[1], Mei-Chu Chung[1] and Wan-Sheng Lo[1,*]

[1]Institute of Plant and Microbial Biology, Academia Sinica, Taipei, Taiwan

[2]Biotechnology Center, National Chung-Hsing University, Taichung, Taiwan

[§]These authors contributed equally to this work
[*]Corresponding author

Supplemental Figures

Figure S1 page 2
Figure S2 page 3
Figure S3 page 4
Figure S4 page 5

Supplemental Tables

Table S1 page 6
Table S2 page 7-8
Table S3 page 9-10
Table S4 page 11-12
Table S5 page 13

Supplemental Figures

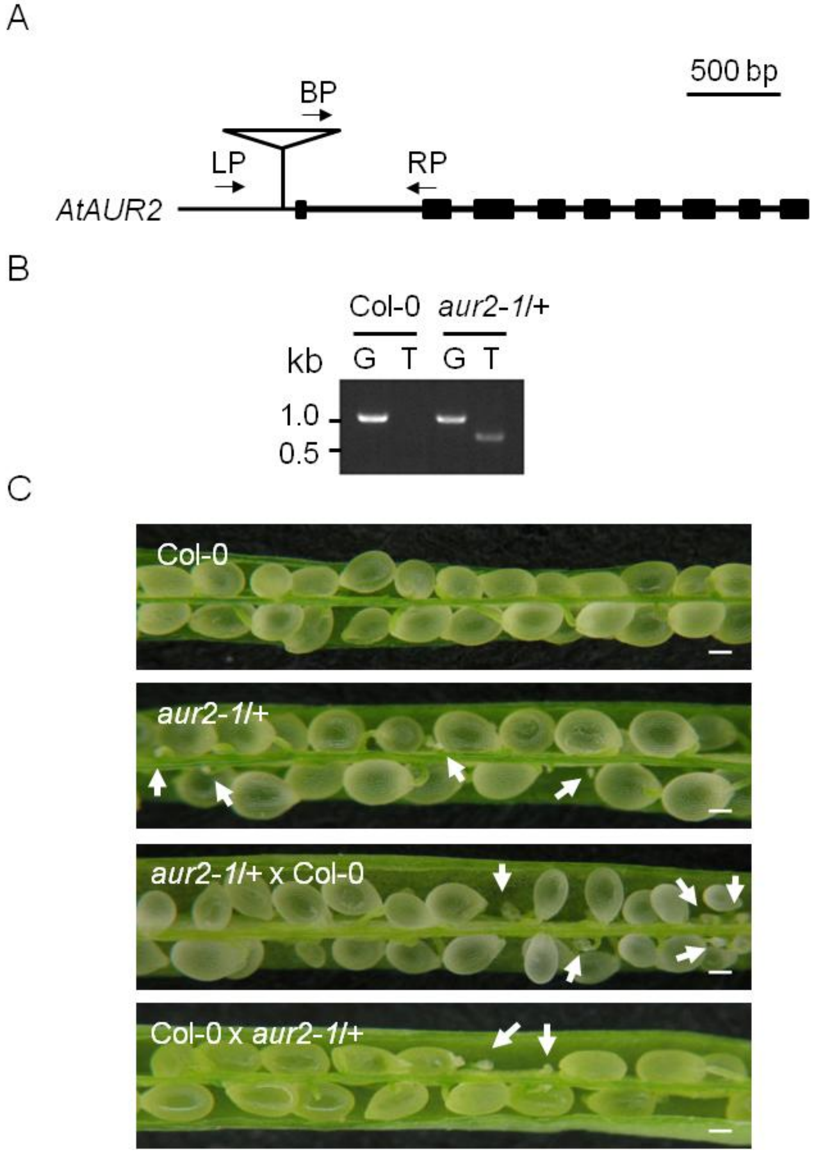

Figure S1. Seed development in self-fertilized and manually crossed *aur2-1*/+.

(A) Schematic representation of T-DNA insertion site in the *AtAUR2* locus. The upstream region is shown as a line and exons as black boxes. The locations of primers (LP and RP) flanking *AtAUR2* and T-DNA–specific primer (BP) are indicated by arrows. **(B)** Genotyping of wild type (Col-0) and *aur2-1*/+. G, PCR product amplified by LP and RP primers for wild type allele; T, amplification by BP and RP primers for T-DNA allele. **(C)** Seed development in the siliques of the self-fertilized wild type (Col-0), *aur2-1*/+ and F1 siliques from reciprocal crosses (female x male). Arrows indicate undeveloped seeds. Scale bar, 200 μm.

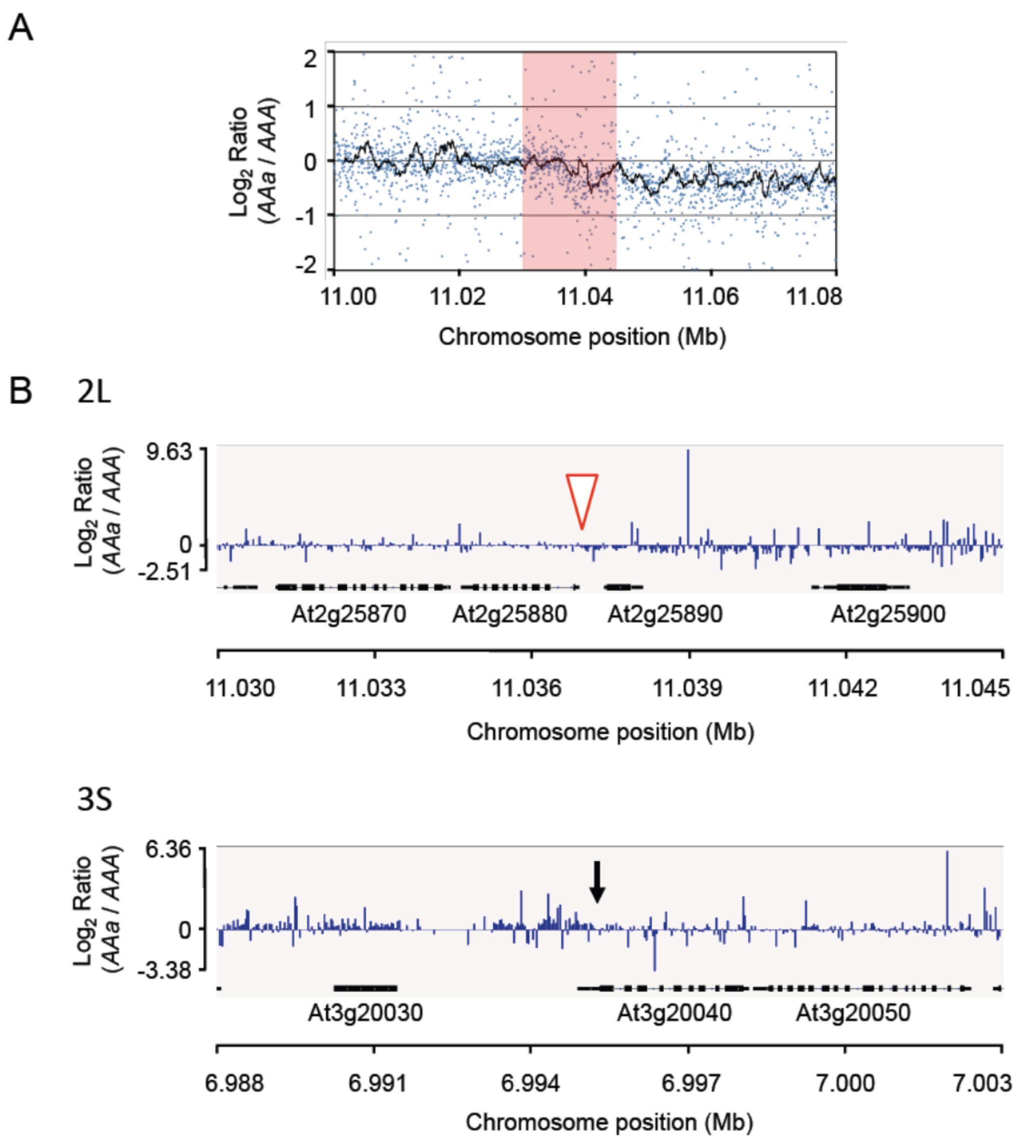

Figure S2. Translocation breakpoints of the tertiary trisomy 2 (*aur2-1*/+; *AAa*).
(A) A zoom-in diagram from the shaded region of the chromosome 2 in the Fig. 2A. The translocation breakpoint is mapped within the shaded region in pink. The moving average of the ratio of signal intensities in 2 trisomy genomes is shown. **(B)** Locations of the *AUR2* locus (At2g25880) with a T-DNA insertion (inverted triangle) and adjacent annotated genes within the shaded region in **(A)** (upper panel). Locations of annotated genes and translocation breakpoint (arrow) on the chromosome 3 (lower panel)

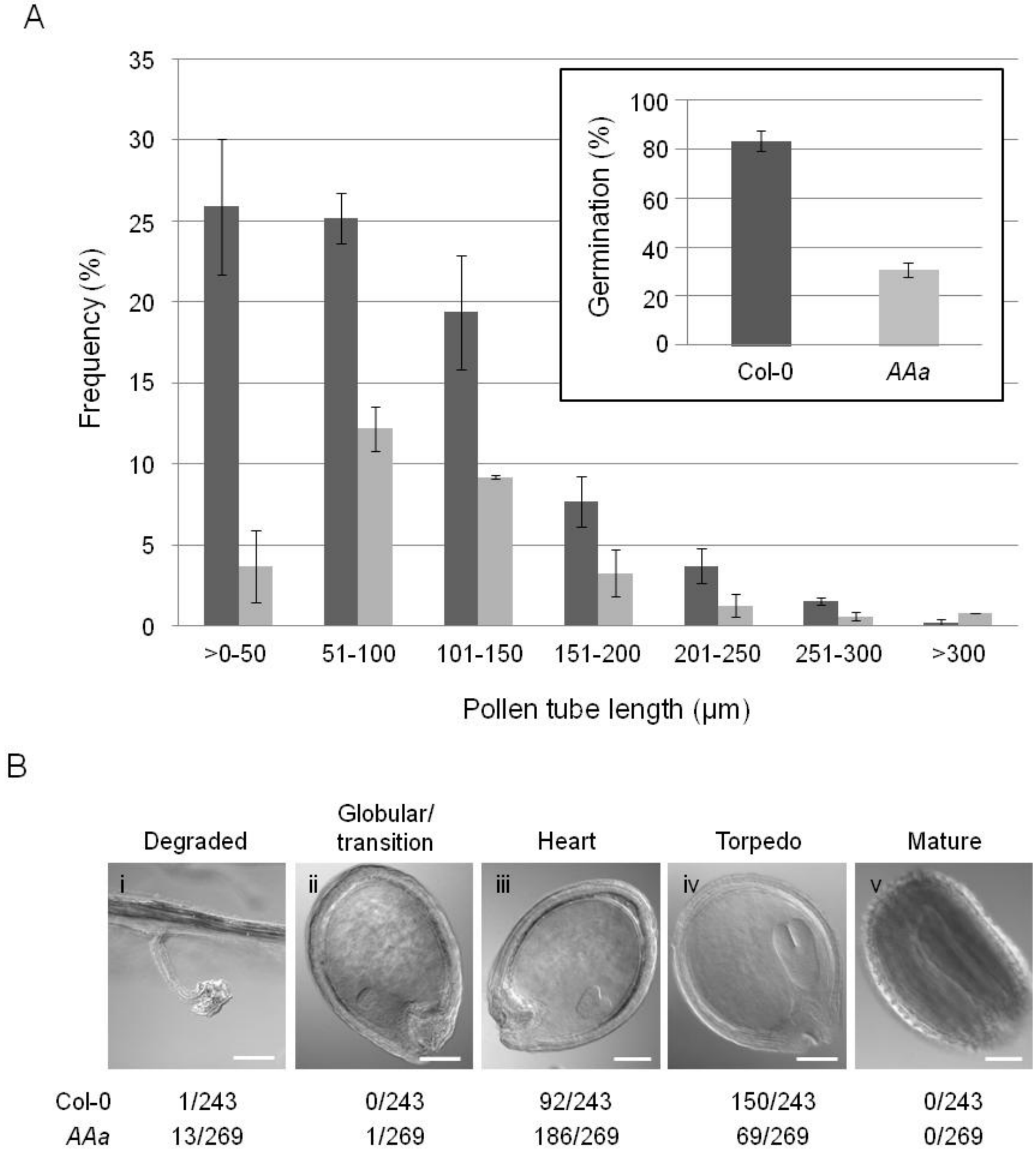

Figure S3. Pollen germination and embryo development in tertiary trisomy 2.
(A) Pollen grains from *aur2-1*/+ show impaired pollen germination and pollen tube growth. The *in vitro* pollen germination of pollen grains from the wild type (Col-0) and *aur2-1*/+ were scored by microscopy and measured by use of ImageJ after 8-h incubation in germination agar medium. Data are mean±SD from 3 independent experiments (n = 400~500). **(B)** Representative differential interference contrast (DIC) images of developing embryos in wild type (Col-0) and tertiary trisomy 2 (*aur2-1*/+; *AAa*) whose ovules were both manually pollinated by WT pollen. Quantitative data of the embryos at 6 days after pollination (DAP) are shown. Scale bar, 100 µm.

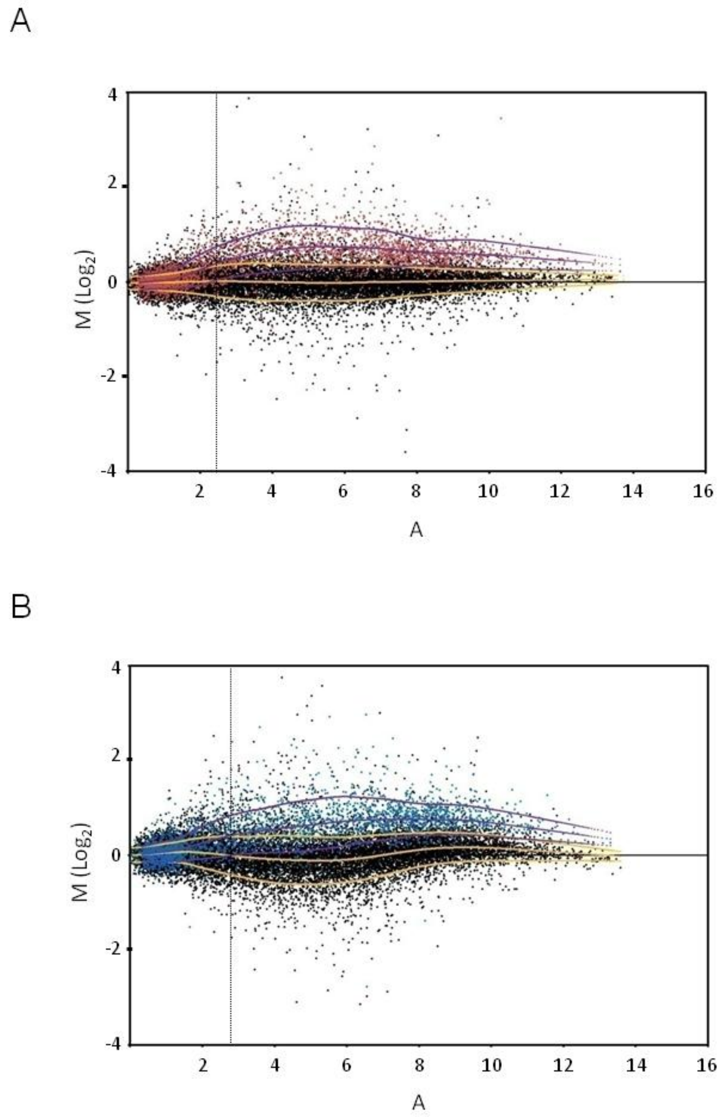

Figure S4. ***M(A)*** **plots of the intensity differences between the trisomics and WT.**
(A) *M(A)* plot of trisomy 2; **(B)** *M(A)* plot of tertiary trisomy 2. The *M* values ($\log_2$ ratios) of the trisomy 2 and wild type (Col-0) are represented in the *y*-axis and the *A* values (the average expression) between the trisomics are represented in the *x*-axis**.** Red and blue dots, entities of the triplicated chromosomes; black dots, entities of other non-triplicated chromosomes; purple and orange lines, moving average. Upper and lower lines of the purple or orange lines, moving average ± SD. Sufficiently strongly expressed transcripts (the dots to the right of the dash lines) were isolated to perform the transcriptional signature analysis in this study. The methodology of data analysis is from Huettel et al. (2008) [5].

Supplemental Tables

Table S1. The ratio of undeveloped seeds in the progeny of self-fertilized and manual crosses of *aur2-1*/+ and wild-type (Col-0) plants

	F1 progeny	
**Parental plants (female x male)**	**Undeveloped seeds (%)**	**Total seeds counted**
Col-0 self-fertilized	0.80	611
aur2-1/+ self-fertilized	18.62	1204
Col-0 × Col-0	1.23	246
aur2-1/+ × Col-0	14.73	325
Col-0 × *aur2-1*/+	8.26	620

Table S2. Enriched gene ontology (GO) terms of genes that are misregulated specifically in the trisomy 2 or tertiary trisomy 2.

Category	GO term	Description (Ontology[b])	p-value	FDR
AAA only	GO:0050896	response to stimulus (P)	6.7E-11	1.9E-08
(up-regulation)	GO:0009813	flavonoid biosynthetic process (P)	1.2E-07	9.3E-06
	GO:0006950	response to stress (P)	4.1E-07	2.1E-05
	GO:0006952	defense response (P)	1.5E-06	6.4E-05
	GO:0019748	secondary metabolic process (P)	6.5E-06	2.5E-04
	GO:0006350	transcription (P)	3.9E-03	5.0E-02
	GO:0007165	signal transduction (P)	8.4E-04	1.4E-02
	GO:0009725	response to hormone stimulus (P)	9.4E-04	1.5E-02
	GO:0003824	catalytic activity (F)	4.8E-06	2.8E-04
	GO:0016740	transferase activity (F)	2.0E-08	2.3E-06
	GO:0003677	DNA binding (F)	2.9E-04	2.1E-02
	GO:0005730	nucleolus (C)	1.3E-04	2.1E-02
AAA only	GO:0009991	response to extracellular stimulus (P)	9.9E-08	4.0E-05
(down-regulation)	GO:0050896	response to stimulus (P)	3.0E-05	3.0E-03
	GO:0009791	post-embryonic development (P)	4.4E-04	2.5E-02
	GO:0006950	response to stress (P)	5.5E-04	2.7E-02
	GO:0016787	hydrolase activity (F)	8.3E-04	4.4E-02
AAa only	GO:0050896	response to stimulus (P)	4.7E-24	3.3E-21
(up-regulation)	GO:0006950	response to stress (P)	1.2E-21	5.1E-19
	GO:0006952	defense response (P)	6.3E-16	1.1E-13
	GO:0051704	multi-organism process (P)	1.3E-15	2.1E-13
	GO:0006955	immune response (P)	8.5E-12	8.5E-10
	GO:0016052	carbohydrate catabolic process (P)	1.2E-09	6.3E-08
	GO:0006810	transport (P)	7.3E-09	3.4E-07
	GO:0006508	proteolysis (P)	2.9E-07	9.6E-06
	GO:0008219	cell death (P)	6.0E-05	1.2E-03
	GO:0015994	chlorophyll metabolic process (P)	5.0E-04	9.0E-03
	GO:0019748	secondary metabolic process (P)	5.0E-04	9.0E-03
	GO:0003824	catalytic activity (F)	2.3E-10	1.0E-07
	GO:0016787	hydrolase activity (F)	4.0E-05	3.1E-03
	GO:0005215	transporter activity (F)	9.0E-05	5.9E-03
	GO:0005773	vacuole (C)	2.5E-12	9.8E-11
	GO:0000502	proteasome complex (C)	1.3E-11	3.3E-10

	GO:0005886	plasma membrane (C)	1.1E-06	1.7E-05
	GO:0005783	endoplasmic reticulum (C)	8.9E-06	1.4E-04
	GO:0009536	plastid (C)	1.90E-03	2.60E-02
	GO:0005739	mitochondrion (C)	2.60E-03	3.40E-02
AAa only	GO:0019758	glycosinolate biosynthetic process (P)	1.8E-19	5.8E-17
(down-regulation)	GO:0005975	carbohydrate metabolic process (P)	1.3E-09	7.6E-08
	GO:0006790	sulfur metabolic process (P)	6.1E-09	3.0E-07
	GO:0016310	phosphorylation (P)	2.6E-08	1.2E-06
	GO:0008152	metabolic process (P)	1.4E-07	5.6E-06
	GO:0022402	cell cycle process (P)	1.3E-06	4.4E-05
	GO:0019748	secondary metabolic process (P)	3.4E-06	1.0E-04
	GO:0042545	cell wall modification (P)	7.6E-05	1.8E-03
	GO:0043687	post-translational protein modification (P)	6.9E-04	1.5E-02
	GO:0003824	catalytic activity (F)	1.3E-18	5.4E-16
	GO:0016740	transferase activity (F)	3.1E-10	6.5E-08
	GO:0016787	hydrolase activity (F)	3.5E-07	4.9E-05
	GO:0016301	kinase activity (F)	4.4E-06	3.1E-04
	GO:0005618	cell wall (C)	3.5E-16	5.9E-14
	GO:0005886	plasma membrane (C)	2.5E-07	8.8E-06
	GO:0043226	organelle (C)	2.9E-03	4.4E-02

[a] GO was analyzed by using agriGO, Du et al. (2012) [33]

[b] Biological process (P); molecular function (F); cellular component (C)

Table S3. Enriched GO terms of significantly upregulated genes in the trisomy 2, tertiary trisomy 2, and trisomy 5

		Trisomy 2 (*AAA*)		Tertiary trisomy 2 (*AAa*)		Trisomy 5	
**GO Term**	**Description**	***p***	**FDR**	***p***	**FDR**	***p***	**FDR**
**GO:0008150**	**Biological_process**						
**GO:0050896**	**response to stimulus**	2.7E-17	2.5E-14	3.4E-49	9.3E-47	2.0E-41	6.7E-39
GO:0006950	response to stress	5.9E-10	7.6E-08	1.4E-37	1.2E-35	3.3E-33	5.7E-31
GO:0009607	response to biotic stimulus	2.7E-08	1.9E-06	7.4E-41	1.0E-38	1.2E-15	1.0E-13
GO:0009628	response to abiotic stimulus	8.1E-06	2.1E-04	5.2E-12	2.0E-10	1.3E-16	1.5E-14
GO:0009719	response to endogenous stimulus	9.5E-10	9.6E-08	1.1E-17	5.2E-16	3.2E-15	2.2E-13
GO:0009605	response to external stimulus	2.1E-10	3.8E-08	1.1E-17	5.2E-16	3.0E-12	1.5E-10
GO:0010033	response to organic substance	1.9E-13	8.4E-11	8.9E-26	1.9E-23	2.1E-30	1.2E-27
GO:0042221	response to chemical stimulus	1.5E-12	4.5E-10	1.4E-27	4.0E-25	1.7E-36	1.8E-33
GO:0009725	response to hormone stimulus	1.2E-04	5.4E-03	6.5E-16	4.0E-14	1.6E-14	2.9E-12
GO:0006952	defense response	1.3E-07	3.3E-06	7.1E-22	7.5E-20	2.1E-09	1.5E-07
GO:0006955	immune response	3.3E-06	6.1E-05	4.5E-18	3.7E-16	6.3E-04	1.5E-02
**GO:0051179**	**localization**	5.4E-03	5.0E-02	2.4E-09	6.6E-08	5.0E-07	1.7E-05
GO:0051234	establishment of localization	6.6E-03	-	3.9E-10	1.2E-08	4.3E-06	1.2E-04
GO:0010876	lipid localization	2.5E-09	2.1E-07	2.0E-03	2.7E-02	4.3E-08	2.7E-06
GO:0006810	transport	6.4E-03	-	3.4E-10	1.2E-08	2.0E-05	1.2E-04
**GO:0050789**	**regulation of biological process**	1.1E-04	1.8E-03	6.1E-05	8.3E-04	1.3E-04	2.7E-03
GO:0051704	multi-organism process	2.4E-06	8.0E-05	1.5E-25	1.0E-23	7.2E-13	4.1E-11
GO:0008152	metabolic process	1.2E-04	1.9E-03	9.8E-03	-	-	-
GO:0044238	primary metabolic process	1.3E-04	1.7E-03	-	-	-	-
GO:0019748	secondary metabolic process	5.6E-08	3.4E-06	6.8E-08	1.7E-06	1.9E-04	3.8E-03
GO:0007165	signal transduction	8.4E-04	1.4E-02	3.5E-03	-	4.6E-05	1.1E-03
GO:0009812	flavonoid metabolic process	9.5E-14	5.1E-12	4.9E-04	7.6E-03	-	-
GO:0016052	carbohydrate catabolic process	-	-	1.2E-09	6.3E-08	-	-
GO:0006508	proteolysis	-	-	2.9E-07	9.6E-06	-	-
GO:0008219	cell death	-	-	2.7E-04	4.9E-03	-	-
GO:0007568	aging	-	-	5.5E-04	8.4E-03	-	-
**GO:0006350**	transcription	3.7E-06	6.6E-05	2.0E-02	-	3.7E-06	6.6E-05
GO:0003700	transcription factor activity	7.6E-06	6.7E-04	-	-	5.6E-07	2.8E-05
GO:0030528	transcription regulator activity	3.1E-05	1.3E-03	-	-	1.3E-06	4.3E-05
**GO:0003674**	**Molecular_function**						
**GO:0003824**	**catalytic activity**	4.2E-07	1.1E-04	6.2E-12	5.6E-10	8.7E-08	8.7E-06
GO:0016740	transferase activity	3.1E-04	2.0E-02	7.8E-03	-	3.6E-04	4.5E-03
GO:0016787	hydrolase activity	2.4E-03	-	1.2E-05	3.6E-04	-	-
GO:0005215	transporter activity	-	-	4.5E-08	2.0E-06	1.8E-05	3.0E-04

GO:0016491	oxidoreductase activity	1.9E-05	1.2E-03	-	-	-	-
GO:0016301	kinase activity	-	-	2.0E-02	-	1.8E-03	0.02
**GO:0005488**	**binding**	8.4E-05	2.8E-03	-	-	3.2E-06	8.0E-05
GO:0003677	DNA binding	1.3E-04	1.3E-04	-	-	1.8E-05	3.0E-04
GO:0005515	protein binding	-	-	-	-	4.1E-05	5.9E-04
GO:0008289	lipid binding	1.0E-02	-	-	-	2.4E-03	0.05
GO:0019825	oxygen binding	3.1E-05	1.3E-03	-	-	-	-
**GO:0005575**	**Cellular_component**						
**GO:0044464**	**cell part**	1.8E-03	0.05	5.8E-07	2.5E-05	1.0E-14	4.6E-13
**GO:0044424**	**intracellular part**	-	-	2.4E-04	3.4E-03	6.6E-08	2.0E-06
GO:0005622	intracellular	-	-	5.3E-04	6.9E-03	5.7E-08	2.0E-06
GO:0005737	cytoplasm	-	-	5.4E-06	1.7E-04	4.1E-04	5.7E-03
GO:0005773	vacuole	-	-	9.8E-06	2.5E-04	3.6E-05	5.9E-04
GO:0016020	membrane	3.0E-02	-	3.9E-08	5.1E-06	1.3E-15	1.1E-13
GO:0005886	plasma membrane	3.0E-02	-	1.1E-05	2.5E-04	9.0E-19	1.6E-16
GO:0005783	endoplasmic reticulum	-	-	6.0E-05	9.8E-04	2.0E-04	3.1E-03
GO:0022624	proteasome accessory complex	-	-	1.3E-11	3.3E-10	-	-
**GO:0030312**	**external encapsulating structure**	7.6E-05	9.1E-03	9.9E-04	9.2E-03	-	-
GO:0005618	cell wall	2.9E-04	2.0E-02	8.7E-04	8.7E-03	-	-
**GO:0043226**	**organelle**	-	-	1.2E-02	-	8.7E-06	1.6E-04

This list includes the upregulated genes common to all three trisomic mutants (1.5× fold change, $p<0.05$). GO was analyzed by use of agriGO, Du et al. (2012) [33]. FDR: chi-square test; - : not detected. Data for trisomy 5 are from Huettel et al. (2008) [5].

Table S4. Enriched GO terms of significantly downregulated genes in the trisomy 2, tertiary trisomy 2, and trisomy 5

		Trisomy 2 (*AAA*)		Tertiary trisomy 2 (*AAa*)		Trisomy 5	
**GO Term**	**Description**	***p***	**FDR**	***p***	**FDR**	***p***	**FDR**
**GO:0008150**	**Biological_process**						
**GO:0008152**	**metabolic process**	-	-	8.4E-09	1.3E-06	2.9E-14	9.3E-12
GO:0044238	primary metabolic process	-	-	7.6E-06	7.6E-04	3.5E-10	1.6E-08
GO:0019748	secondary metabolic process	-	-	1.3E-05	8.8E-04	-	-
GO:0044237	cellular metabolic process	-	-	1.3E-05	8.8E-04	1.7E-05	4.2E-04
GO:0019538	protein metabolic process	-	-	2.0E-03	-	2.5E-05	5.8E-04
**GO:0009987**	**cellular process**	-	-	4.6E-04	1.0E-02	3.1E-13	3.3E-11
GO:0044237	cellular metabolic process	-	-	1.5E-05	5.6E-04	2.6E-13	3.3E-11
GO:0034641	cellular nitrogen compound metabolic process	-	-	1.4E-04	4.3E-03	5.7E-15	2.9E-12
GO:0009058	biosynthetic process	-	-	2.4E-03	-	2.5E-12	1.7E-10
GO:0043436	oxoacid metabolic process	-	-	1.4E-04	4.3E-03	7.7E-10	7.2E-08
GO:0006519	cellular amino acid and derivative metabolic process	-	-	-	-	9.0E-08	3.3E-06
GO:0019761	glucosinolate biosynthetic process	-	-	4.1E-25	1.4E-22	-	-
GO:0044262	cellular carbohydrate metabolic process	1.0E-02	-	1.0E-22	2.7E-20	2.0E-02	-
GO:0022402	cell cycle process	-	-	6.9E-04	1.5E-02	-	-
GO:0042254	ribosome biogenesis	-	-	-	-	6.5E-19	9.8E-16
GO:0006412	translation	-	-	-	-	3.0E-08	1.2E-06
GO:0034660	ncRNA metabolic process	-	-	-	-	1.8E-05	7.8E-04
GO:0015979	photosynthesis	-	-	-	-	3.6E-06	9.7E-05
**GO:0050896**	**response to stimulus**	9.5E-14	2.8E-11	1.0E-03	4.0E-02	4.0E-02	-
GO:0006950	response to stress	6.4E-05	2.3E-03	1.0E-02	-	-	-
**GO:0032502**	**developmental process**	-	-	-	-	2.7E-04	4.0E-03
GO:0009791	post-embryonic development	4.0E-02	-	-	-	2.6E-12	1.7E-10
GO:0003006	reproductive developmental process	-	-	-	-	2.4E-04	3.8E-03
**GO:0003674**	**Molecular_function**						
**GO:0003824**	**catalytic activity**	2.9E-05	2.9E-03	6.3E-17	5.4E-15	1.0E-12	4.7E-11
GO:0016787	hydrolase activity	3.4E-04	0.02	2.5E-07	1.1E-05	1.2E-06	3.7E-05
GO:0004091	carboxylesterase activity	2.0E-06	4.3E-04	3.8E-06	1.7E-04	-	-
GO:0016491	oxidoreductase activity	1.0E-02	-	1.1E-18	1.7E-16	5.1E-03	-

GO:0016740	transferase activity	-	-	5.0E-06	1.2E-04	1.0E-02	-
GO:0016301	kinase activity	-	-	1.3E-04	1.9E-03	3.0E-02	-
**GO:0005575**	**Cellular_component**						
**GO:0044464**	**cell part**	-	-	1.1E-11	3.6E-10	1.2E-24	1.4E-23
GO:0044424	intracellular part	-	-	1.1E-03	2.0E-02	4.0E-34	1.3E-32
GO:0005622	intracellular	-	-	4.2E-03	-	3.4E-34	1.3E-32
GO:0005737	cytoplasm	-	-	8.0E-03	-	1.7E-44	9.6E-43
GO:0016020	membrane	-	-	1.7E-03	3.0E-02	1.0E-05	6.8E-05
GO:0005886	plasma membrane	-	-	6.7E-04	3.0E-02	7.2E-05	4.3E-04
GO:0005730	nucleolus	-	-	-	-	2.3E-08	1.7E-07
**GO:0005576**	**extracellular region**	-	-	1.6E-04	4.3E-03	1.0E-02	5.0E-02
GO:0005618	cell wall	-	-	3.5E-15	3.8E-13	1.7E-03	9.3E-03
**GO:0043226**	**organelle**	-	-	-	-	2.6E-32	7.3E-31

This list includes the downregulated genes common to all three trisomic mutants (1.5× fold change, $p<0.05$). GO was analyzed by use of agriGO, Du et al. (2012) [33]. FDR: chi-square test; - : not detected. Data for trisomy 5 are from Huettel et al. (2008) [5].

Table S5. The Punnett square for modeling segregation of the self-fertilized tertiary trisomy 2 (*AAa*, *aur2-1*/+)

♀ \ ♂	1/6 *AA*	2/6 *Aa*	2/6 *A*	1/6 *a*
1/6 *AA*	1/36 *AAAA*	2/36 *AAAa*	2/36 *AAA*	1/36 *AAa*
2/6 *Aa*	2/36 *AAAa*	4/36 *AAaa*	4/36 *AAa*	2/36 *Aaa*
2/6 *A*	2/36 *AAA**	4/36 *AAa*	4/36 *AA*	2/36 *Aa*
1/6 *a*	1/36 *AAa*	2/36 *Aaa*	2/36 *Aa*	1/36 *aa*

A, wild-type *AUR2*; *a*, *aur2-1*; shaded boxes, no gametophytes or offspring; *, progeny expected with low occurrence in the offspring.

Ratio indicates theoretical segregation for gametophytes and progeny.
